# Tracking Hyperpolarized [1‐^13^C] Pyruvate and [1‐^13^C] L‐Lactate Metabolism in the Healthy and Post‐Stroke Mouse Brain

**DOI:** 10.1002/nbm.70094

**Published:** 2025-07-06

**Authors:** Thanh Phong Lê, Lara Buscemi, Mario Lepore, Elise Vinckenbosch, Bernard Lanz, Rolf Gruetter, Lorenz Hirt, Jean‐Noël Hyacinthe, Mor Mishkovsky

**Affiliations:** ^1^ Laboratory of Functional and Metabolic Imaging École polytechnique fédérale de Lausanne (EPFL) Lausanne Switzerland; ^2^ Department of Clinical Neurosciences Lausanne University Hospital (CHUV) Lausanne Switzerland; ^3^ Department of Fundamental Neurosciences University of Lausanne (UNIL) Lausanne Switzerland; ^4^ CIBM Center for Biomedical Imaging Lausanne Switzerland; ^5^ Animal Imaging and Technology École polytechnique fédérale de Lausanne (EPFL) Lausanne Switzerland; ^6^ Image Guided Intervention Laboratory Faculty of Medicine, University of Geneva Geneva Switzerland

## Abstract

Tracking hyperpolarized (HP) ^13^C labeling from either [1‐^13^C] pyruvate or [1‐^13^C] lactate is a useful tool to assess intermediary metabolism in vivo, which has already been translated from preclinical to clinical research. HP [1‐^13^C] pyruvate and [1‐^13^C] lactate provide complementary views on the same metabolic pathway, and both have been tested as potential neuroprotective agents in the context of acute brain injuries, with more convincing evidence for a beneficial effect of lactate. Our aim here was to investigate and compare HP [1‐^13^C] pyruvate and [1‐^13^C] lactate performance as metabolic contrast agents in the brains of healthy mice and mice subjected to middle cerebral artery occlusion, a model of ischemic stroke. We analyzed the metabolite ratios and quantified the real‐time apparent kinetic rates of their cerebral metabolism. We found that the cerebral metabolism of both HP [1‐^13^C] pyruvate and HP [1‐^13^C] lactate showed significant alterations after transient cerebral ischemia in mice, reflecting the damage as well as the metabolic reprogramming set in motion to meet the energetic demands in the acute phase of stroke. There was a significant decrease in metabolite ratios (cLPR, cAPR for pyruvate bolus and cPLR, cALR for lactate bolus) and kinetic rates (c*k*_PL_ for pyruvate bolus and c*k*_LP_ for lactate bolus). These values progressively decreased from sham to 1 h and 2 h after reperfusion measurements. Overall, while pyruvate is better established as an imaging probe, and lactate appears advantageous on the therapeutic side, both bring information to interrogate brain metabolism in physiological and pathophysiological conditions in real time. This study prepares the ground for further investigation to fully exploit the potential of HP metabolic contrasts for stroke theranostics.

## Graphical abstract

Hyperpolarized [1‐^13^C] pyruvate and [1‐^13^C] lactate provide complementary views on the same metabolic pathways. We investigated their performance as cerebral metabolic contrast agents in healthy mice and mice subjected to transient ischemia. We found that the cerebral metabolism of both substrates was significantly altered after ischemia and discuss the potential of these substrates as theranostic biosensors for stroke.

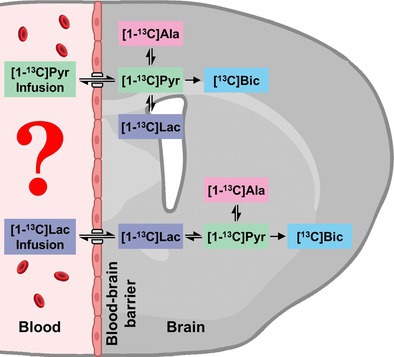

## 1 Introduction

Magnetic resonance spectroscopy (MRS) is a non‐invasive tool for studying in vivo metabolism, widely used in oncology and neuroimaging [1, 2, 3, 4]. With the production of biocompatible solutions incorporating hyperpolarized (HP) metabolic precursors, new MRS applications have emerged that allow the interrogation of intermediary metabolism with higher sensitivity and time resolution [5, 6], bringing a novel strategy for real‐time metabolic imaging [7, 8]. Although many metabolites can be HP using different techniques [9, 10], due to the excellent properties of [1‐^13^C] pyruvate for hyperpolarization and being a key molecule at important metabolic crossroads, the majority of preclinical HP MR research has employed [1‐^13^C] pyruvate [11], making it the first HP ^13^C‐labelled metabolic contrast agent translated into clinical studies [12, 13]. In fact, [1‐^13^C] pyruvate is anticipated to become a next‐generation molecular imaging tool for cancer and is currently being evaluated in over 50 clinical trials, as reported on ClinicalTrials.gov. The reduction of [1‐^13^C] pyruvate to [1‐^13^C] lactate by lactate dehydrogenase (LDH) is widely probed in health and disease [7, 8, 13]. Another important MRS marker of [1‐^13^C] pyruvate metabolism is [^13^C] bicarbonate, generated after the entry of pyruvate into the TCA cycle through the pyruvate dehydrogenase (PDH) complex [7, 8, 13].

Using a bolus of HP [1‐^13^C] lactate instead of HP [1‐^13^C] pyruvate gives an alternative view of this metabolic network. Indeed, solutions containing HP [1‐^13^C] lactate were employed to investigate LDH and PDH activities in vivo in various organs [14, 15, 16, 17] including the brain [18, 19]. The physiological concentration of blood lactate is substantially higher than that of pyruvate; thus, a typical bolus injection of HP lactate does not greatly alter its circulating concentration. Lactate is an interesting molecule for neuroimaging, as it was shown to contribute to cerebral energy metabolism [20, 21, 22]. Interestingly, various observations indicate that lactate can be taken up by neurons and transformed, via LDH, into pyruvate to fuel mitochondrial respiration [18]. Considering that administering HP [1‐^13^C] pyruvate or HP [1‐^13^C] lactate provides a means to measure the exchange between the two metabolites with different directionality, the first aim of the present study was to compare the metabolic outcome of each substrate in the naive mouse brain.

Independently of their potential utility as HP metabolic contrast agents, pyruvate and lactate have been studied as prospective neuroprotective compounds. Ischemic stroke is the second [23] leading cause of mortality worldwide and the main cause of disability in adults [24]. Recanalization within a narrow time window by intravenous thrombolysis and/or mechanical clot removal is the only currently available treatment option in the acute phase of ischemic stroke [25, 26, 27]. Several recent trials have extended the time window for thrombectomy in carefully selected patients up to 24 h from symptom onset [28, 29]. Even with these treatments, the global burden of stroke remains high. Therefore, numerous preclinical studies aiming to improve the outcome after stroke have tested neuroprotective strategies targeted at recovering suffering cerebral tissue in the acute phase of ischemic stroke [30, 31]. Among them, the administration of pyruvate [32, 33, 34] or lactate [35, 36, 37, 38, 39] after stroke was investigated at the preclinical level, with lactate resulting in a beneficial reduction in the infarct size and improvement of the neurological outcome, while pyruvate appeared less protective than lactate in some studies [35, 40]. Furthermore, lactate administration was shown to improve glucose availability in patients with acute brain injuries [41] and an exploratory randomized placebo‐controlled clinical trial of lactate administration is currently underway in patients with acute ischemic stroke [42]. With the prior assumption of their potential as neuroprotective agents and their implementation as HP metabolic contrast agents, the second aim of this work was to investigate the performances of [1‐^13^C] pyruvate and [1‐^13^C] lactate as HP biosensors in the transient middle cerebral artery occlusion (MCAO) stroke model.

The comparisons comprise (1) model‐free analysis of HP contrasts based on metabolite ratios calculated from the summed ^13^C MRS spectra and (2) metabolic models to quantify the apparent kinetic rates and thus evaluate the dynamics of HP [1‐^13^C] pyruvate and HP [1‐^13^C] lactate metabolism, both in sham‐operated and stroke mice. Finally, in the context of a potential therapeutic use, the injected dose of either pyruvate or lactate in this study (1 mmol/kg) is rather high compared to the typical concentrations of HP tracers (≈0.10 mmol/kg with pyruvate [12] and ≈0.06 mmol/kg with lactate [43]); we used ^1^H MRS in the healthy animals to assess whether the high doses led to any significant modification of the neurochemical profiles.

## 2 Experimental

### 2.1 Animal Experimentation

All experimental procedures with mice were approved by the regulatory body of the Canton of Vaud, Switzerland (Service de la Consommation et des Affaires Vétérinaires), license numbers VD2017.5 and VD2017.6, were conducted according to federal and local ethical guidelines, and complied with the ARRIVE guidelines. Male C57BL/6J mice (6–10 weeks, Charles River, France) were maintained in an animal facility with controlled humidity and temperature, with a 12 h light/dark cycle and free access to food and water.

### 2.2 Study Design

**FIGURE 1:**
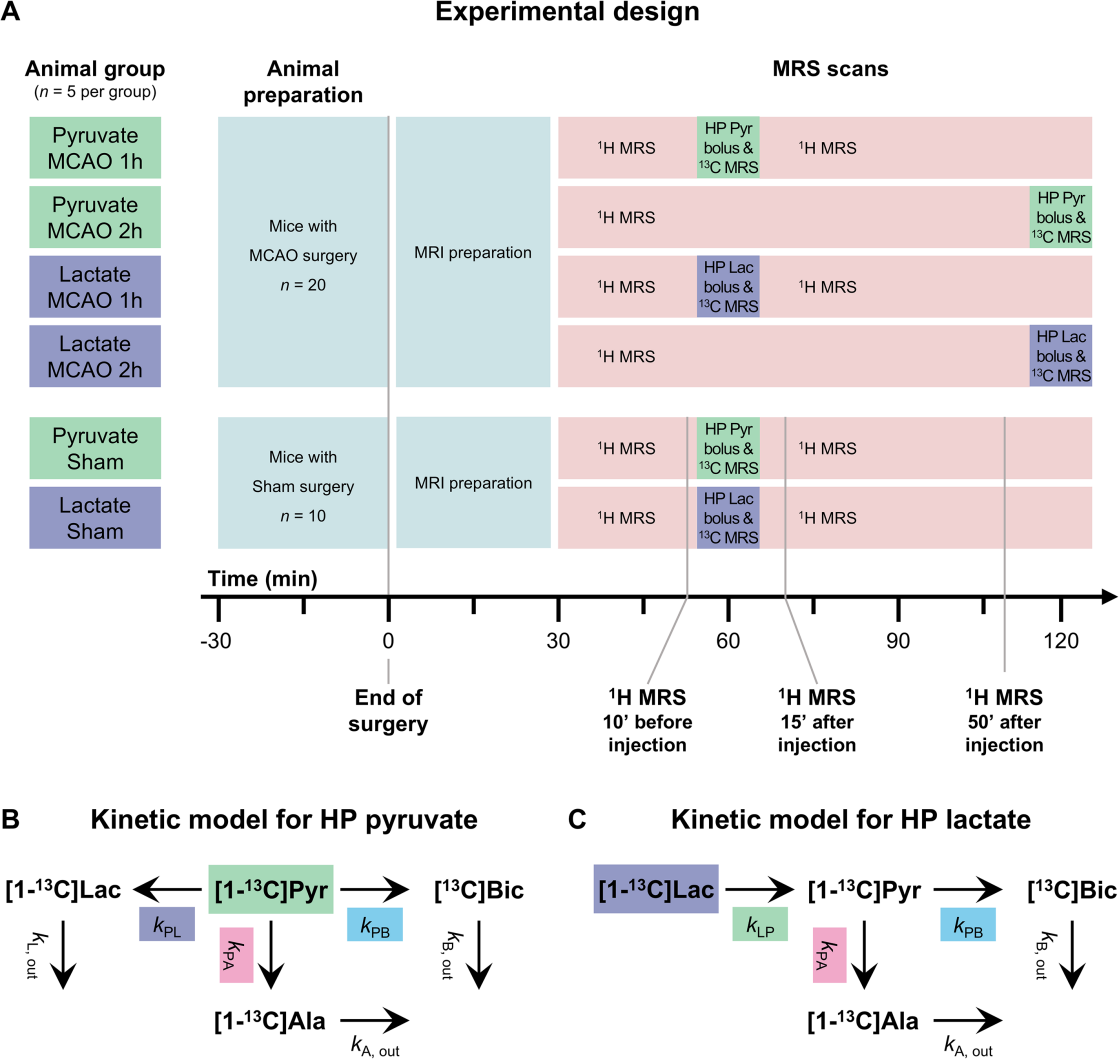
(A) Outline of the experimental design for the HP ^13^C and ^1^H MRS experiments. This study includes six groups: MCAO mice with HP [1‐^13^C] pyruvate injection at either 1 h or 2 h after reperfusion; MCAO mice with HP [1‐^13^C] lactate injection at either 1 h or 2 h after reperfusion; sham mice with injection of either HP [1‐^13^C] lactate or HP [1‐^13^C] pyruvate at 1 h after surgery. After MRI preparation, the ^1^H MRS neurochemical profile of the left striatum is continuously measured (apart from when ^13^C MRS after HP substrate injection is acquired) and compared in sham animals between 10 min before injection and 15 min after injection (the last ^1^H MRS datapoint before, and first after ^13^C HP MRS, respectively) and between 10 min before injection and 50 min after injection (the last ^1^H MRS measurement before ^13^C HP MRS and last ^1^H MRS measurement of the session) after surgery. (B–C) Schematics of cerebral transport and metabolism of HP [1‐^13^C] pyruvate (B) and HP [1‐^13^C] lactate (C) used for mathematical kinetic modeling.

The global cerebral metabolism of HP [1‐^13^C] pyruvate and [1‐^13^C] lactate was measured in C57BL/6J mice subjected to either sham or transient MCAO surgery. Mice were kept under anesthesia with 1.5%–2.0% isoflurane in 60% oxygen from the beginning of the surgery to the end of the MR measurements. An outline of the experimental design is summarized in Figure 1A. Briefly, each animal received a single bolus of either HP pyruvate or HP lactate for HP ^13^C MRS. Mice that underwent transient MCAO surgery received an intravenous injection of HP substrate at either 1 h or 2 h after reperfusion (i.e., four groups: MCAO 1 h pyruvate, MCAO 2 h pyruvate, MCAO 1 h lactate, and MCAO 2 h lactate). Animals that underwent sham surgery (controls) received the HP substrate injection 1 h after surgery only (i.e., two groups: sham lactate and sham pyruvate). Overall, 30 mice (*n* = 5 per group) were included in this study.

Localized ^1^H MRS spectra were acquired in the striatum of the left (ipsilateral) hemisphere of each mouse. ^1^H MRS spectra collected 10 min before, and 15 min and 50 min after the HP bolus injection in sham animals were compared for changes associated with the high dose administered. In mice that underwent MCAO surgery, besides proton spectroscopy, T_2_W images were acquired to verify that the surgery led to an ischemic lesion.

At the end of the experiment, mice were sacrificed while still under the effect of anesthesia by cervical dislocation followed by decapitation.

### 2.3 Transient MCAO Model of Stroke

A focal lesion in the left brain hemisphere was induced by transient cerebral ischemia as previously described [35, 44]. In summary, the neck was incised, and both left common and external carotid arteries were exposed and ligated. A silicone‐coated nylon monofilament (Doccol Corp., Sharon, USA) was inserted through the common carotid artery and gently driven into the internal carotid artery until reaching the middle cerebral artery. The occluding filament was removed after 30 min to allow blood flow restoration. During occlusion, the left femoral vein was cannulated to allow the intravenous injection of the HP solution. Laser‐Doppler flowmetry was used to monitor the regional cerebral blood flow (rCBF) during the surgery through a flexible probe (Perimed AB, Sweden) glued to the skull at 1 mm posterior and 6 mm lateral from bregma. The intervention was considered successful if the rCBF remained below 20% of the baseline during occlusion and increased above 50% of the initial value within 10 min of reperfusion. Sham‐operated mice followed a similar surgical procedure without suture insertion or artery ligation.

### 2.4 Hyperpolarization

For HP pyruvate experiments, 25 μL of neat [1‐^13^C] pyruvic acid (Sigma Aldrich, Buchs, Switzerland) doped with 21 mM of OX063 trityl radical (Albeda Research, Værløse, Denmark) were prepared in the form of frozen beads. To neutralize the acid upon dissolution, frozen beads of 10 M NaOH in D_2_O were added to the sample cup at a 1:1.44 (v/v) ratio.

For HP lactate experiments, 100 μL of sodium L‐[1‐^13^C] lactate (Sigma Aldrich) in water/glycerol doped with 25 mM of OX063 radical was prepared in the form of frozen beads.

The samples were loaded and HP in a 7T/1K DNP polarizer [45]. A liquid‐state polarization of 59.0% ± 5.0% was reported in this system for pyruvate [46], and of 33.1% ± 8.9% for lactate. To note, we used the trityl radical to single out the inherent metabolic changes induced by stroke, avoiding any influence of the polarizing agent, as can happen with the use of the nitroxyl radical TEMPOL that we used in our previous report [19], where we found it facilitated the conversion of lactate to pyruvate [47].

### 2.5 Magnetic Resonance Measurements

All MR measurements were performed on a 9.4 T/31 cm horizontal actively shielded magnet (Magnex Scientific, Abingdon, UK) equipped with a Varian INOVA spectrometer (Varian, Palo Alto, USA). Upon reperfusion, mice were transferred into the MRI scanner with a home‐built ^1^H quadrature/^13^C single loop coil above the head, whose sensitivity profile was reported in a previous study [19].

### 2.6 Proton Imaging

Anatomical axial T_2_ weighted (T_2_W) images were acquired with a fast multi‐slice spin‐echo sequence (effective echo time TE_eff_ = 52 ms, TR = 4000 ms, 18 × 9 mm^2^ FOV, 256 × 128 matrix) at the beginning of the MR scan to provide localization for the shimming voxels, as well as within 5 min of the HP injection and at 125 min after reperfusion to assess the evolution of the lesion.

### 2.7 Proton Spectroscopy

Localized proton spectroscopy was acquired on the same animals in which the HP substrate was injected, except for one animal in each lactate group. Static field inhomogeneity was corrected using the FASTESTMAP routine [48, 49] in a 2.0 × 1.8 × 2.0 mm^3^ voxel located in the striatum, where the ischemic lesion was expected. ^1^H MRS was performed using the SPECIAL pulse sequence [50] (TE = 2.8 ms, TR = 4000 ms, 200 ms acquisition time, and 10 blocks of 16 scans) and repeated continuously up to 120 min after reperfusion or after surgery, except for the time required for shimming, HP ^13^C MRS, and ^1^H imaging. The absolute metabolite concentrations were quantified using LCModel V6.3‐1N [51]. Values with Cramer–Rao lower bounds (CRLBs) above 40% were discarded from further analysis.

### 2.8 HP ^13^C MRS

To optimize the spectral resolution, static field inhomogeneity was corrected in a 3.6 × 6.9 × 4.5 mm^3^ voxel within the brain using the FASTESTMAP routine [48, 49]. HP substrate injections were performed at 1 h or 2 h after reperfusion in MCAO mice and at 1 h after surgery in sham‐operated animals via the femoral vein using an automated protocol [45]. Each animal received a single bolus of either HP [1‐^13^C] pyruvate or [1‐^13^C] lactate. ^13^C MR spectra were immediately acquired every 3 s with a 30° BIR‐4 adiabatic pulse‐acquire sequence. The injection volume was set to 450 μL, including 125 μL of dead volume. The choice of the bolus concentration was based on the protective doses reported in Yi et al. [32] and Berthet et al. [36] for pyruvate and lactate, respectively. The actual doses received were measured after the experiment by high‐resolution NMR, being 1.12 ± 0.13 mmol/kg for HP pyruvate and 1.08 ± 0.19 mmol/kg for HP lactate (mean ± SD).

### 2.9 Data Processing

#### 2.9.1 ^13^C Metabolite Ratios

The signals from the first 120 s after injection were summed, the metabolite peaks fitted, and the corresponding areas under the curves (AUCs) were calculated using the Bayesian Data‐Analysis Software Package V4.01 (Washington University in St. Louis). The peak areas of [1‐^13^C] lactate, [1‐^13^C] alanine, [1‐^13^C] pyruvate, and [^13^C] bicarbonate were then used to compute the metabolite ratios. To reduce the variability between individuals, the ratios were normalized to the HP infusate dose, specifically by multiplying the ratio by the amount of HP pyruvate or lactate injected and dividing by the animal's body weight.

#### 2.9.2 ^13^C Kinetic Modeling

For each animal, the time course of [1‐^13^C] lactate, [1‐^13^C] alanine, [1‐^13^C] pyruvate, and [^13^C] bicarbonate signals was built by fitting each individual spectrum using the Bayesian Data‐Analysis Software Package and plotting the corresponding peak integral as a function of time.

To quantify the apparent kinetic rates from the labeled metabolite time courses, multi‐compartment models describing the metabolic kinetics after HP [1‐^13^C] lactate or HP [1‐^13^C] pyruvate bolus (Figure 1B,C) injections were derived from their respective simplified schemes of cerebral transport and metabolism (Supplementary information Figure S1) and using the following assumptions:

- Each conversion or elimination step was modeled as a first‐order chemical reaction.
- A single apparent pyruvate‐to‐lactate turnover step (pyruvate model) or lactate‐to‐pyruvate turnover step (lactate model) was used to describe both the transport of the substrate across the blood–brain barrier (BBB) and LDH exchange. The corresponding kinetic rate constant is related rather to the transport step, which is slower compared to the LDH interconversion step [18, 52].
- Although the LDH inter‐conversion step is reversible, only the forward reaction was considered given that the bolus injection shifts the equilibrium towards downstream metabolites. The forward kinetic rate values were found similarly in models taking the reversible LDH exchange into account (Supplementary information Figure S2).
- Conversion of [1‐^13^C] pyruvate to [^13^C] bicarbonate was accounted for in a single step since the intermediate product, ^13^CO_2_, was not detectable due to the limited bandwidth of radio‐frequency pulses.
- Elimination steps (efflux rate) were added to the end products.

The dynamic ^13^C MRS signal is a combination of the uptake of the precursor, the metabolic conversion into downstream metabolites, and the signal losses resulting from the longitudinal spin–lattice relaxation and repeated RF excitations. The time evolution of the downstream metabolites in the multicompartment models shown above can then be described using a set of differential equations.

Following an injection of HP pyruvate:

$$\frac{d}{\mathrm{dt}}\left(\begin{array}{c} \mathrm{Lac}\\ \mathrm{Ala}\\ \mathrm{Bic} \end{array}\right)\left(t\right)=\left(\begin{array}{ccc} -k_{\text{L,}\;\text{out}}-F-\frac{1}{T_{1}} & 0 & 0\\ 0 & -k_{\text{A,}\;\text{out}}-F-\frac{1}{T_{1}} & 0\\ 0 & 0 & -k_{\text{B,}\;\text{out}}-F-\frac{1}{T_{1}} \end{array}\begin{array}{c} \;k_{\mathrm{PL}}\\ {\begin{array}{c} \;\\ \;\\ \;k \end{array}}_{\mathrm{PA}}\\ \;{\begin{array}{c} \;\\ \;\\ k \end{array}}_{\mathrm{PB}} \end{array}\right)\left(\begin{array}{c} \mathrm{Lac}\\ \mathrm{Ala}\\ \mathrm{Bic}\\ \mathrm{Pyr} \end{array}\right)\left(t\right)$$

Lac(t), Ala(t), Bic(t), and Pyr(t) denote the time‐varying signal amplitude of the corresponding HP ^13^C metabolites; *k*_X_, X ∈ {PL; PA; PB; A, out; B, out; L, out} denote the first‐order kinetic rate constants for the conversion or elimination of metabolites, as summarized in Figure 1B. F is the term accounting for the loss of magnetization resulting from the RF pulse:

$$F=-\frac{\ln\left(\cos\left(\alpha \right)\right)}{\mathrm{TR}}$$

Where α = 30° is the flip angle of the RF pulse applied with a repetition time TR = 3 s. Finally, T_1_ is the longitudinal relaxation time constant, approximated to 15 s for all metabolites in a similar range to previously reported values [53, 54]. While varied within a range of 10 to 40 s, the parameter T_1_ smoothly changed the calculated kinetic rates. However, the differences between groups remained mostly similar (Supplementary information Figure S3).

By assuming a negligible contribution from endogenous ^13^C metabolites due to their low thermal polarization and natural abundance, the following initial conditions can be set:

$$\left(\begin{array}{c} \mathrm{Lac}\\ \mathrm{Ala}\\ \mathrm{Bic} \end{array}\right)\left(t=0\right)=\left(\begin{array}{c} 0\\ 0\\ 0 \end{array}\right)$$

Similarly, the following set of differential equations is derived for the metabolism following injection of HP lactate:

$$\frac{d}{\mathrm{dt}}\left(\begin{array}{c} \mathrm{Pyr}\\ \mathrm{Ala}\\ \mathrm{Bic} \end{array}\right)\left(t\right)=\left(\begin{array}{ccc} -k_{\mathrm{PA}}-k_{\mathrm{PB}}-F-\frac{1}{T_{1}} & 0 & 0\\ k_{\mathrm{PA}} & -k_{\text{A,}\;\text{out}}-F-\frac{1}{T_{1}} & 0\\ k_{\mathrm{PB}} & 0 & -k_{\text{B,}\;\text{out}}-F-\frac{1}{T_{1}} \end{array}\begin{array}{c} \;k_{\mathrm{LP}}\\ \begin{array}{c} \;\\ \;\\ \;0 \end{array}\\ \;\begin{array}{c} \;\\ \;\\ 0 \end{array} \end{array}\right)\left(\begin{array}{c} \mathrm{Pyr}\\ \mathrm{Ala}\\ \mathrm{Bic}\\ \mathrm{Lac} \end{array}\right)\left(t\right)$$

With the following initial conditions:

$$\left(\begin{array}{c} \mathrm{Pyr}\\ \mathrm{Ala}\\ \mathrm{Bic} \end{array}\right)\left(t=0\right)=\left(\begin{array}{c} 0\\ 0\\ 0 \end{array}\right)$$

Where Pyr(t), Ala(t), Bic(t) and Lac(t) denote the time‐varying signal amplitude of the corresponding HP ^13^C metabolites, *k*_X_, X ∈ {LP; PA; PB; A, out; B, out} the first‐order kinetic rate constants for the conversion between the metabolites or their elimination, as summarized in Figure 1C.

The time course of the ^13^C signal intensity of the injected metabolite, either lactate or pyruvate, was fitted with a polynomial function and used as an input function for solving the set of differential equations. Then, apparent kinetic rate constants were determined by fitting each model to the corresponding experimental ^13^C signal time course using a trust‐region least squares algorithm in a routine implemented in MATLAB R2023b (MathWorks, Natick, USA). Finally, to account for experimental variability in body weight and substrate concentration, the kinetic rates corresponding to the metabolic step originating from the injected metabolite were additionally normalized to the injected dose.

### 2.10 Statistical Analysis

We performed statistical analyses using the MATLAB R2023b software (MathWorks, Natick, USA). To compare ^13^C MRS data, one‐way analysis of variance (one‐way ANOVA) was used, followed by Tukey–Kramer’s test to correct for multiple comparisons, while repeated measures Kruskal–Wallis tests were used in the analysis of ^1^H MRS data. A *p*‐value below 0.05 was considered statistically significant. All data are presented as mean ± standard deviation. Based on previous reports [55, 56], we evaluated the linearity between the metabolite ratio and the apparent kinetic rate of each metabolic step by calculating the Pearson correlation coefficient (*ρ*) in MATLAB R2023b.

## 3 Results

**FIGURE 2:**
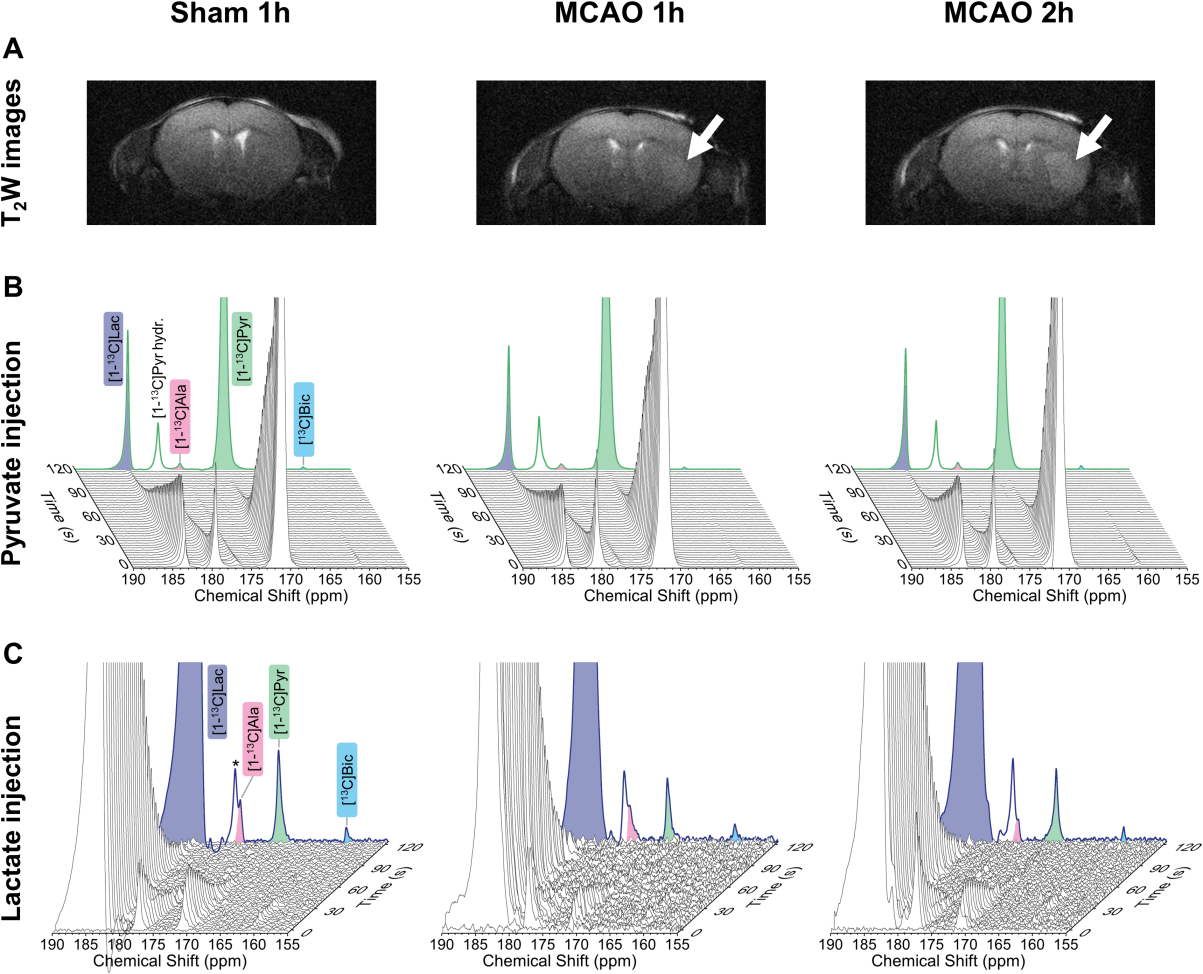
Typical data acquired in healthy controls (sham 1 h after surgery) and mice after middle cerebral artery occlusion (MCAO) 1 h or 2 h after reperfusion. (A) Representative axial T_2_W images acquired in the brain using an FSEMS sequence (four averages). Arrows indicate the injured region resulting from the induced transient ischemic stroke. No lesion is observed in sham. Already slightly noticeable at 1 h, the lesion becomes readily visible and delimited by 2 h after reperfusion. (B–C) Representative dynamic ^13^C MRS spectra acquired from healthy and stroke mouse brains following infusion of (B) HP [1‐^13^C] pyruvate (lb = 10 Hz) or (C) HP [1‐^13^C] lactate (lb = 20 Hz). The bold colored spectra are the sum of the first 120 s after infusion. The peak at 177.7 ppm (*) is an impurity in the lactate stock solution.

As expected, normal brain structure was observed in anatomical T_2_W images of sham (healthy) animals (Figure 2A, left panel). In mice that underwent 30 min transient cerebral ischemia, slight morphological modifications could be seen at 1 h after reperfusion (Figure 2A, center panel), and the typical striatal lesion was readily visible at 2 h after reperfusion (Figure 2A, right panel).

Representative dynamic cerebral HP ^13^C MRS spectra acquired in sham and MCAO animals are shown in Figure 2B,C. When HP [1‐^13^C] pyruvate was infused, it was converted into HP [1‐^13^C] lactate, HP [1‐^13^C] alanine, and HP [^13^C] bicarbonate. HP [1‐^13^C] pyruvate hydrate was observed as well and represents about 8% of the [1‐^13^C] pyruvate signal amplitude. Alternatively, when HP [1‐^13^C] lactate was infused, it was converted into HP [1‐^13^C] pyruvate, which was then further converted into either HP [1‐^13^C] alanine or HP [^13^C] bicarbonate. The peak visible at 177.7 ppm is a known chemical impurity from the lactate stock solution and not a result of mouse metabolism (confirmed by room temperature HR NMR measurements, data not shown).

**FIGURE 3:**
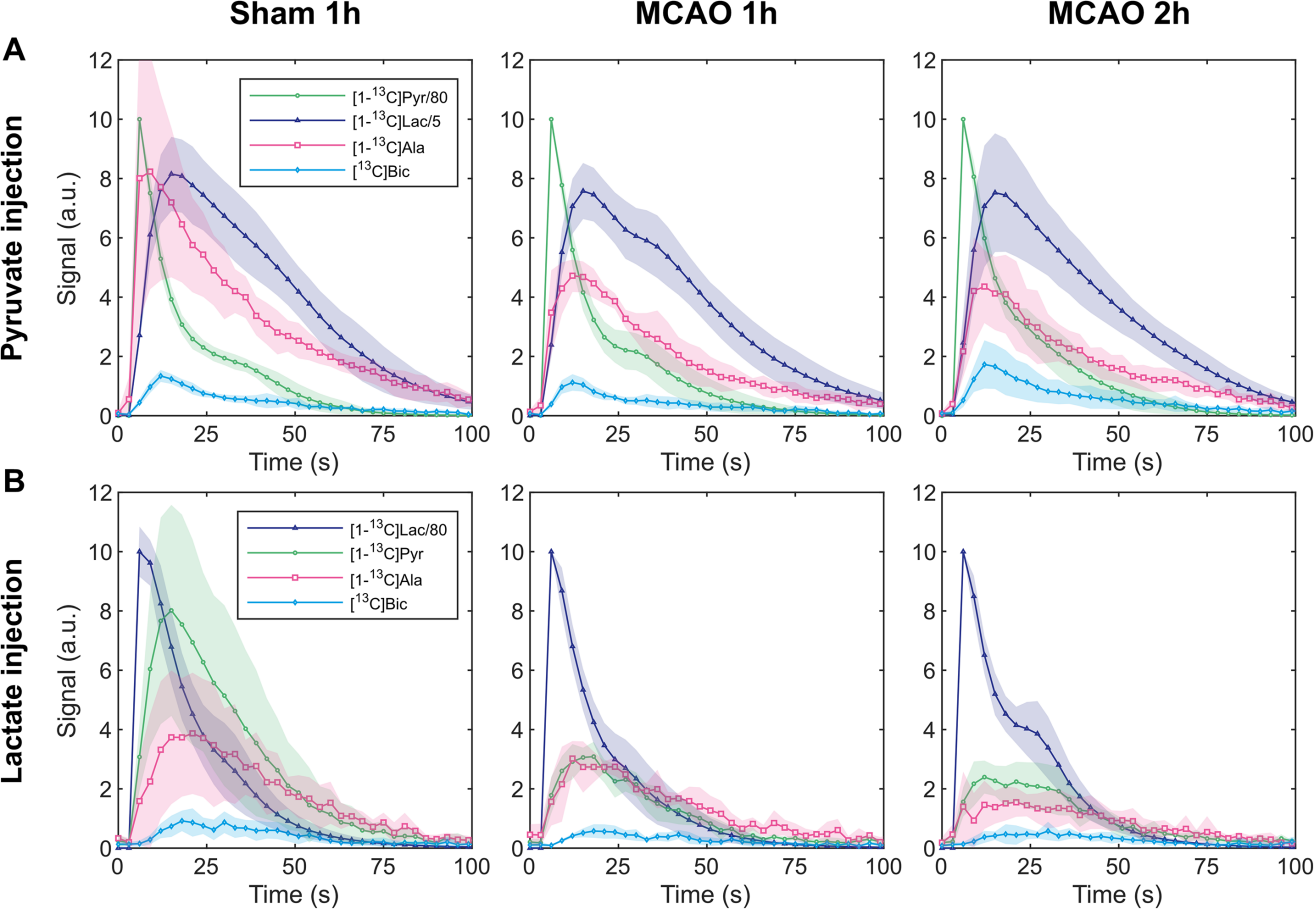
Metabolic time courses acquired following injection of HP [1‐^13^C] pyruvate (A) or [1‐^13^C] lactate (B) averaged across all experiments within each group (*n* = 5 mice per group). The signal intensity represents the fitted peak integral of a given metabolite and time point. For each individual experiment, the data were normalized beforehand to the highest signal intensity data point, set to an arbitrary unit value of 10. The shaded areas represent the standard deviation of the mean. The pyruvate and lactate signals were scaled for display purposes as indicated in the legend.

The average time course for the individual HP ^13^C metabolite signals for sham and MCAO mice is reported in Figure 3. In experiments with an HP [1‐^13^C] pyruvate bolus, a subtle decrease in the labeling of [1‐^13^C] lactate and a strong reduction in [1‐^13^C] alanine labeling were observed after stroke compared to sham (Figure 3A). Conversely, when HP [1‐^13^C] lactate was given as a bolus, a distinct decrease in the labeling of [1‐^13^C] pyruvate was readily visible in all stroke mice compared to healthy animals (sham). The [1‐^13^C] alanine and [^13^C] bicarbonate labeling was also lower, but to a lesser extent (Figure 3B).

**FIGURE 4:**
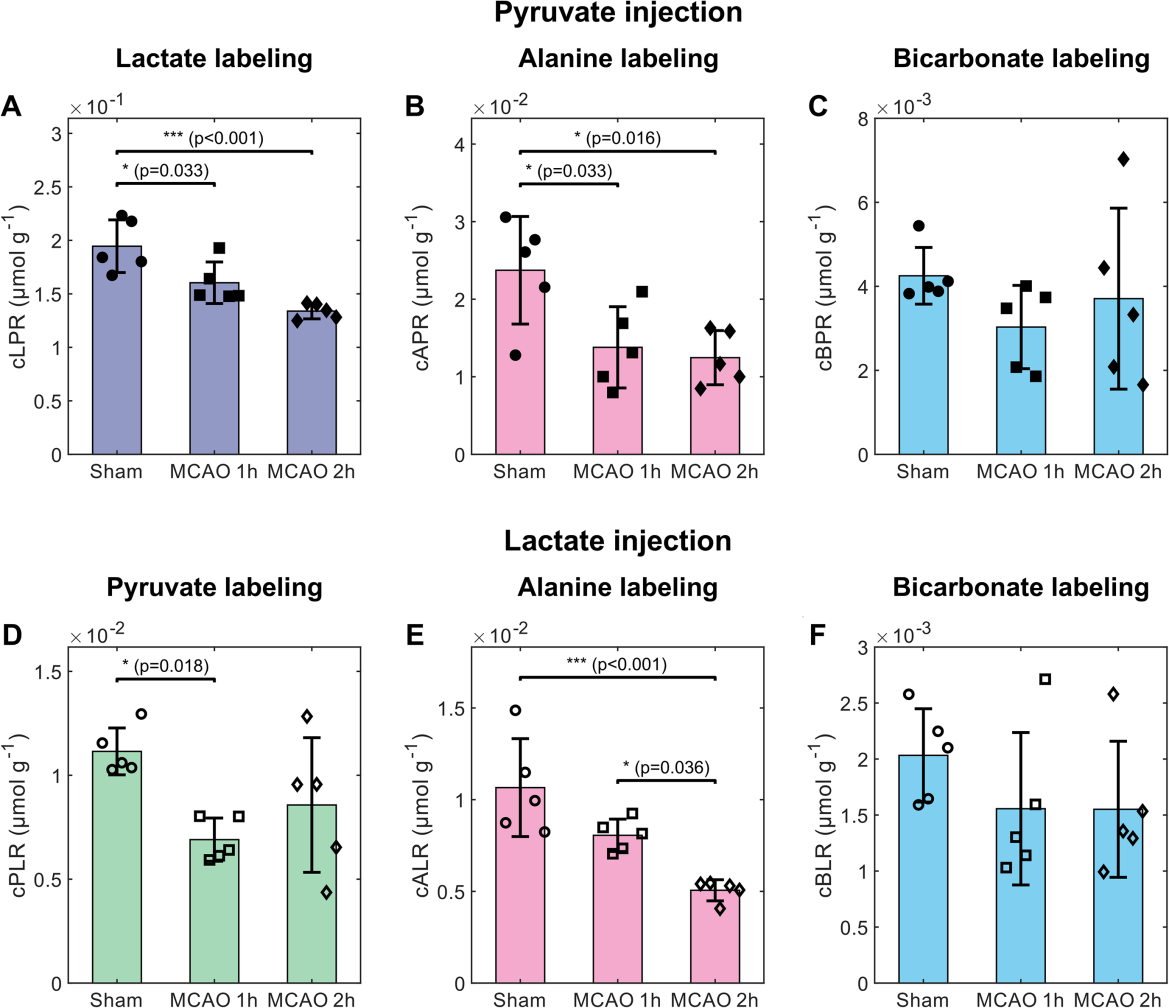
Normalized metabolite ratios in healthy sham and MCAO animals following injection of HP [1‐^13^C] pyruvate (A–C) or HP [1‐^13^C] lactate (D–F). Data are displayed as the mean ± standard deviation and overlaid with individual data points (circles: sham, squares: MCAO 1 h, diamonds: MCAO 2 h, filled shapes: pyruvate injection, hollow shapes: lactate injection). The lactate‐to‐pyruvate ratio (cLPR, A), alanine‐to‐pyruvate ratio (cAPR, B), bicarbonate‐to‐pyruvate ratio (cBPR, C), pyruvate‐to‐lactate ratio (cPLR, D), alanine‐to‐lactate ratio (cALR, E), bicarbonate‐to‐lactate ratio (cBLR, F) are all normalized to the actual [1‐^13^C] pyruvate or [1‐^13^C] lactate dose injected.

The metabolite ratio analysis, from the summed spectra within 120 s of injection, further highlights differences in substrate metabolic conversions between shams and animals subjected to MCAO following a pyruvate or lactate bolus (Figure 4). After pyruvate infusion, the normalized lactate‐to‐pyruvate ratio (cLPR) was found to be significantly different between healthy and both groups of stroke animals (18% and 31% reduction at 1 h and 2 h after reperfusion, respectively, Figure 4A). The alanine labeling was similar for both MCAO timepoints and significantly lower than sham by 42% in the 1 h after MCAO mice and by 48% in the 2 h after MCAO mice (cAPR, Figure 4B). No significant changes were observed in bicarbonate labeling after stroke (cBPR, Figure 4C). Changes in the metabolite labeling were also found after HP lactate infusion. The normalized pyruvate‐to‐lactate ratio (cPLR) was significantly lower by 38% 1 h after reperfusion compared to sham (Figure 4D). Further differences were found in the alanine labeling, with a normalized alanine‐to‐lactate ratio (cALR) that was lowest in the MCAO 2 h group, which was significantly reduced by 37% and 53% compared to the MCAO 1 h and the sham groups, respectively (Figure 4E). As with HP pyruvate infusion, no significant changes were observed in bicarbonate labeling after stroke (cBLR, Figure 4F).

**FIGURE 5:**
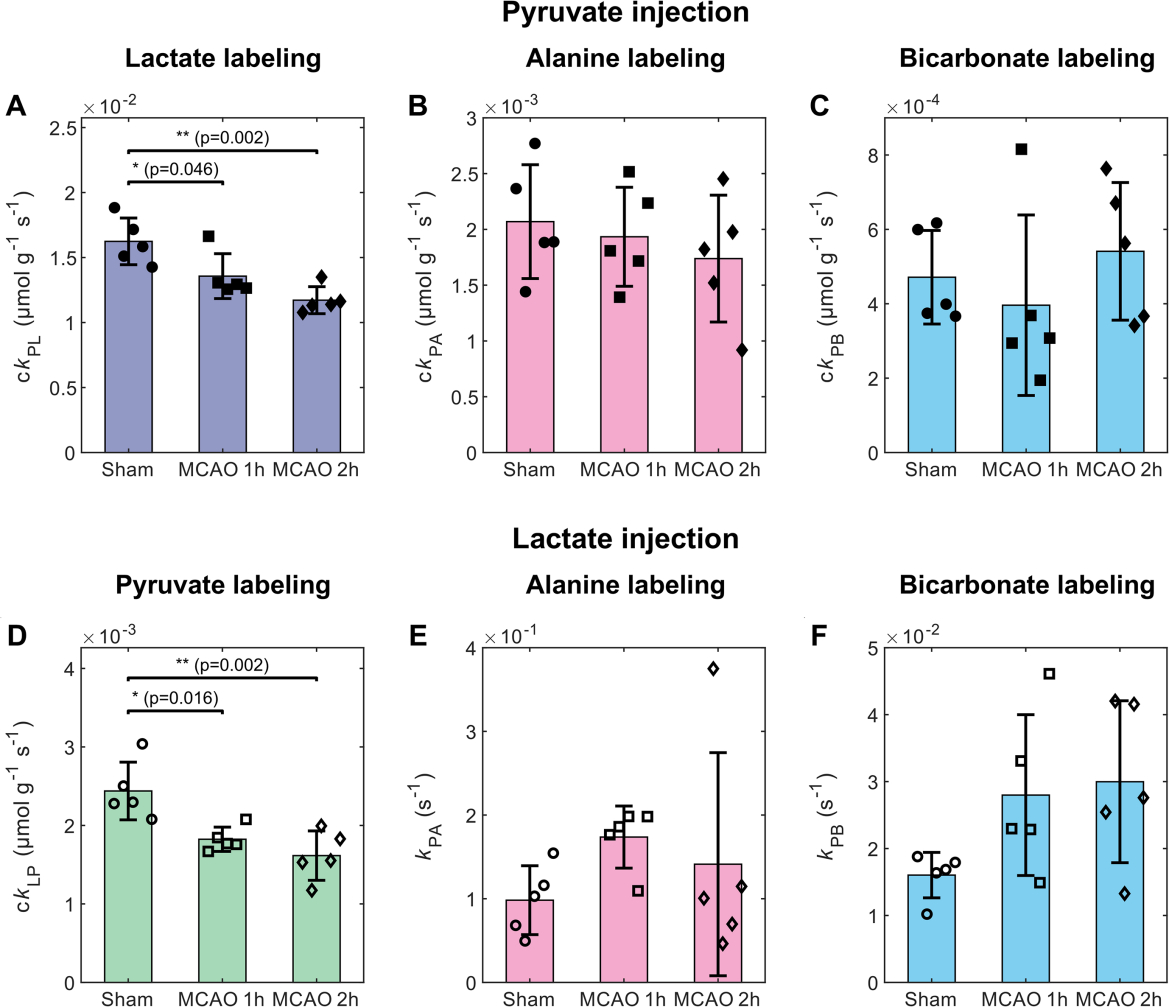
Kinetic rates following injection of HP [1‐^13^C] pyruvate (A–C) or HP [1‐^13^C] lactate (D–F). Data are displayed as the mean ± standard deviation and overlaid with individual data points (circles: sham, squares: MCAO 1 h, diamonds: MCAO 2 h, filled shapes: pyruvate injection, hollow shapes: lactate injection). Normalized rate constants of pyruvate‐to‐lactate (c*k*_PL_, A), pyruvate‐to‐alanine (c*k*_PA_, B), pyruvate‐to‐bicarbonate (c*k*_PB_, C), lactate‐to‐pyruvate (c*k*_LP_, D) conversion. Rate constants of pyruvate‐to‐alanine (*k*_PA_, E) and pyruvate‐ to‐bicarbonate (*k*_PB_, F) turnover.

We then quantified the kinetics of HP pyruvate and HP lactate cerebral metabolism using mathematical modeling. In HP pyruvate experiments, the kinetic rate of pyruvate‐to‐lactate conversion was significantly slower in stroke animals than in sham, both at 1 h (17%) and 2 h (28%) after reperfusion (c*k*_PL_, Figure 5A). In HP lactate experiments, the apparent kinetic rate of lactate‐to‐pyruvate labeling was significantly reduced in stroke animals at 1 h (25%) and 2 h (34%) after reperfusion (c*k*_LP_, Figure 5D), compared to sham. The dynamics of alanine labeling (c*k*_PA_ and *k*_PA_, Figure 5B,E) were not statistically different between healthy and stroke mice for both HP substrates. Interestingly, while the dynamics of bicarbonate labeling were similar across the three groups with a bolus of HP pyruvate (c*k*_PB_, Figure 5C), the average kinetic rates of pyruvate‐to‐bicarbonate conversion after an HP lactate bolus were almost doubled in stroke animals compared to sham, although we did not observe statistically significant differences (*p*‐values of 0.19 and 0.11 when comparing sham to MCAO 1 h and 2 h respectively, *k*_PB_, Figure 5F). The remaining kinetic parameters related to these models are available in the Supplementary information Figures S5 and S6.

**FIGURE 6:**
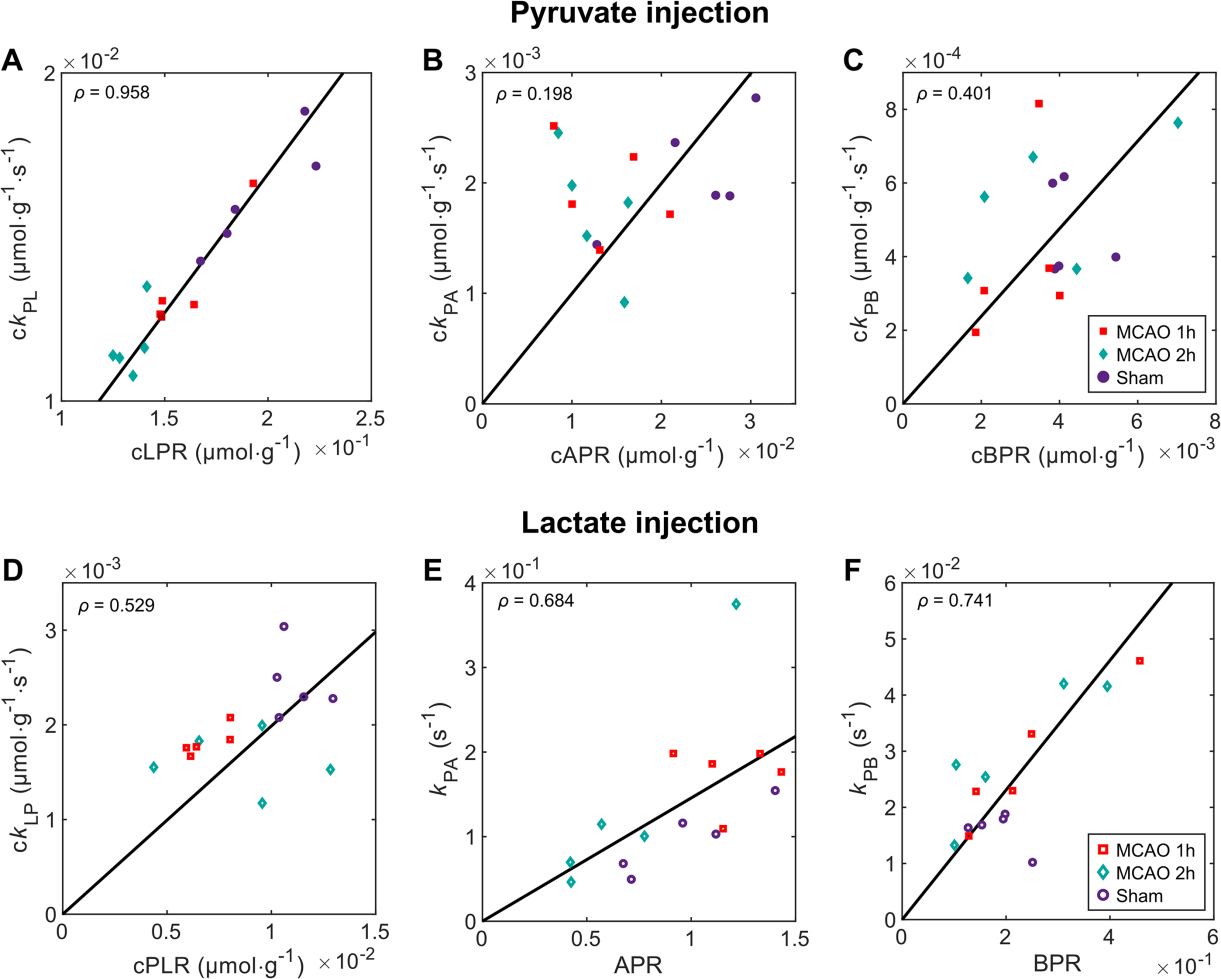
Correlation between kinetic rate constants (modeling) and metabolite ratios (model‐free), for HP [1‐^13^C] pyruvate (A–C) and [1‐^13^C] lactate (D–F) experiments. For the lactate to alanine or bicarbonate metabolism, we used the ratio to pyruvate, since pyruvate is the substrate pool of the corresponding metabolic rate in the kinetic model (i.e., the starting and ending points are the same as in the kinetic model). The bold line represents the linear least squares fit. The Pearson correlation coefficient (*ρ*) is displayed in the top‐left corner of each plot.

We then tested the correlation between model‐free analysis (metabolite ratios) and model‐based kinetic analysis (Figure 6). In the case of HP [1‐^13^C] pyruvate injection, the model‐free analysis and the kinetic modeling were very strongly linearly correlated for the lactate labeling (*ρ* = 0.958), whereas in the case of HP [1‐^13^C] lactate injection only a moderate linear correlation was found in the pyruvate labeling (*ρ* = 0.529). We further observed that the correlation for both alanine and bicarbonate labeling was substantially stronger when injecting HP lactate than HP pyruvate (*ρ* = 0.684 compared to 0.198 for alanine and *ρ* = 0.741 compared to *ρ* = 0.401 for bicarbonate). Note that for the alanine and bicarbonate labeling in HP lactate experiments, we calculated metabolite ratios with respect to the pyruvate signal such that the starting and ending points were identical to the corresponding kinetic rate in the model (Supplementary information Figure S4).

**FIGURE 7:**
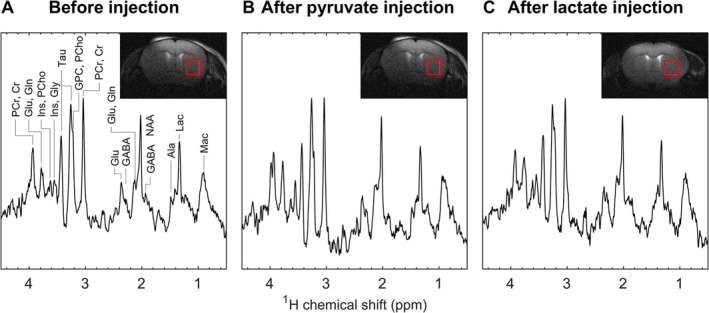
Representative ^1^H MRS spectra (160 averages, 640 s of acquisition time for each point) and voxel position in the striatum of sham mice (A) 10 min before HP ^13^C MRS, (B) 15 min after an HP [1‐^13^C] pyruvate bolus, (C) 15 min after an HP [1‐^13^C] lactate bolus. 5 Hz of line broadening was applied to each spectrum for visualization purposes. The red rectangles indicate the spectroscopy voxel within the striatum. Abbreviations: alanine (Ala), creatine (Cr), γ‐aminobutyric acid (GABA), glutamine (Gln), glutamate (Glu), glycine (Gly), glycerophosphocholine (GPC), myo‐inositol (Ins), lactate (Lac), macromolecules (Mac), N‐acetyl‐aspartate (NAA), phosphocholine (PCho), phosphocreatine (PCr), and taurine (Tau).

The neurochemical profiles of healthy animals were quantified from ^1^H MRS to test for changes induced by the pyruvate or lactate bolus. Representative proton spectra are shown in Figure 7. For each individual animal, the absolute concentration estimates of selected metabolites at 15 and 50 min after the bolus were compared to the baseline before the bolus (Table 1, with data from individual animals in Supplementary information Figure S7). We found that even at the high doses used in this study, metabolite concentrations at both time points after the bolus were not significantly different from baseline values. The evolution of the neurochemical profile in this mouse stroke model has been well characterized [57], the most remarkable change being the increase in lactate concentration. In a previous publication, we reported endogenous metabolite changes under similar experimental conditions but without the administration of a bolus of HP tracer [19]. Proton spectra acquired using the current setup indicated comparable changes in MCAO mice that received a bolus of either pyruvate or lactate (Supplementary information Figures S8 and Figure S9).

**TABLE 1:** Concentration estimates in [μmol/g] of selected metabolites detected in the striatum of sham animals at 10 min before injection, 15 min after injection, and 50 min after injection of either HP [1‐^13^C] pyruvate or HP [1‐^13^C] lactate. No significant difference was observed between before and after bolus concentrations.

Metabolites	Mice with HP [1‐^13^C] pyruvate injection			Mice with HP [1‐^13^C] lactate injection		
	10′ before bolus	15′ after bolus	50′ after bolus	10′ before bolus	15′ after bolus	50′ after bolus
Gln	3.3 ± 2.0	3.8 ± 2.2	4.0 ± 1.3	3.8 ± 2.0	3.7 ± 2.0	3.5 ± 2.0
Glu	4.7 ± 1.4	4.6 ± 0.8	4.9 ± 1.4	4.2 ± 0.9	4.7 ± 0.7	4.6 ± 1.7
Lac	5.8 ± 0.9	7.1 ± 0.8	6.7 ± 1.2	5.5 ± 1.4	5.3 ± 1.8	5.6 ± 1.3
NAA	5.0 ± 0.6	5.5 ± 0.3	5.7 ± 0.7	4.7 ± 0.7	4.4 ± 0.7	4.4 ± 0.8
NAA + NAAG	5.2 ± 0.5	5.5 ± 0.3	6.0 ± 0.5	5.2 ± 1.0	4.8 ± 0.6	4.9 ± 0.6
Glu + Gln	8.1 ± 2.8	8.4 ± 2.0	8.9 ± 2.0	8.0 ± 1.5	8.4 ± 1.3	8.1 ± 2.1
Cr + PCr	8.1 ± 0.3	8.3 ± 1.2	8.5 ± 1.2	7.6 ± 1.2	7.5 ± 0.8	9.2 ± 0.8
Ins	4.8 ± 1.1	4.7 ± 0.9	5.5 ± 1.5	5.2 ± 1.3	4.8 ± 0.8	5.4 ± 0.6

Abbreviations: creatine (Cr), glutamine (Gln), glutamate (Glu), lactate (Lac), myo‐inositol (Ins), N‐acetyl‐aspartate (NAA), N‐acetyl‐aspartyl‐glutamate (NAAG), phosphocreatine (PCr).

## 4 Discussion

The HP ^13^C label inter‐conversion step between pyruvate and lactate is a useful tool to assess in vivo metabolism, starting either with labeled pyruvate or lactate. HP [1‐^13^C] pyruvate and HP [1‐^13^C] lactate provide complementary views on the same metabolic network, with different entry points and directions. Here, we compared their cerebral metabolism quantitatively in similar experimental conditions. We investigated metabolism after stroke in the MCAO mouse model using sham mice as a control, while administering substrates at doses matching their presumed neuroprotective doses (i.e., 1 mmol/kg), which are rather high compared to the typical doses of HP tracers. Nonetheless, based on the proton spectroscopy analysis, it is important to highlight that neither the high bolus of pyruvate nor that of lactate induced significant changes in the metabolite pool size in the striatum of healthy brains at early (15 min) or late (50 min) time points after the HP substrate bolus.

To analyze their performance as HP contrast agents and to account for their similarities and differences, we compared their metabolic evolution using a model‐free analysis using metabolite ratios, and their metabolic dynamics with kinetic modeling. For HP pyruvate, we used the accepted metabolic kinetic model. For HP lactate metabolism, we developed a new model that allowed us to quantify the metabolic dynamics of lactate for the first time. After a bolus of either substrate, the uptake is rate‐limited by transport across the BBB [18, 52, 58]. Consequently, the HP substrate signal mostly originates from the blood [18, 52, 59], while downstream metabolite signals are expected to arise within brain tissue (Supplementary information Figure S1). Note that here the brain is simplified and considered as a single tissue compartment, as signal measurement does not allow differentiating between brain cellular compartments (i.e., neurons, glial cells, vascular and mural cells). With this approach, in HP [1‐^13^C] pyruvate experiments, all the steps measured via ^13^C MRS stem directly from pyruvate and can thus be interpreted as a combination of transport across the BBB and metabolism. On the other hand, in HP [1‐^13^C] lactate experiments, only the measured pyruvate results from a combination of lactate transport across the BBB and its enzymatic conversion to pyruvate in the brain. The subsequent steps from pyruvate into alanine or bicarbonate are considered as intracellular processes, since the precursor was generated in the brain tissue. The metabolite ratio analysis in healthy animals when injecting HP pyruvate or lactate shows that the cLPR is one order of magnitude higher than the cPLR, respectively. This is coherent with the relative difference in pool sizes, as lactate has a substantially higher endogenous abundance, approximately 20 times that of pyruvate in healthy animals [60]. The alanine and bicarbonate labeling are of the same order of magnitude for both substrates.

In healthy animals, the kinetic analysis revealed a faster pyruvate‐to‐lactate rate by one order of magnitude compared to the lactate‐to‐pyruvate rate (c*k*_PL_ = (1.6 ± 0.2)·10^−2^ μmol·g^−1^·s^−1^ and c*k*_LP_ = (2.4 ± 0.4)·10^−3^ μmol·g^−1^·s^−1^, respectively). While the permeability of the BBB is higher for lactate than for pyruvate [60], we found a higher c*k*_PL_ than c*k*_LP_. This could be related to the difference between pyruvate and lactate pool sizes and their relative increase upon the administration of the respective HP bolus. In healthy animals, the endogenous pyruvate concentration is about 20 times lower than that of lactate. Consequently, after a bolus administration of equivalent concentrations of the HP ^13^C tracers, the fractional enrichment of ^13^C in the pyruvate pool would be much larger than that in the lactate pool. In the latter case, the metabolism of labeled pyruvate derived from lactate is constrained by its lower fractional enrichment. Moreover, the higher affinity of MCT1 for pyruvate compared to lactate (K_m_ for pyruvate: 0.6–1.0 mM; K_m_ for lactate: 2.2–4.5 mM [61]) further supports this observation.

To compare the label exchange dynamics to alanine and bicarbonate for both substrates, we used the non‐normalized kinetic rates because in HP lactate experiments, they do not directly originate from the injected HP precursor (*k*_PA_ and *k*_PB_ respectively, Supplementary information Figures S5 and, S6). After HP [1‐^13^C] lactate injection, both *k*_PA_ and *k*_PB_ were two orders of magnitude higher than after HP [1‐^13^C] pyruvate injection. As discussed above, the dynamics after the pyruvate bolus include the transport across the BBB of the tracer, which is a rate‐limiting step, thus leading to the slower apparent conversion of pyruvate into alanine and bicarbonate, considering that the essential portion of the measured ^13^C‐pyruvate signal arises from the blood compartment.

Given the need for neuroprotective agents in the context of ischemic stroke and the potential of pyruvate and lactate as HP metabolic biosensors, we also compared their performance as metabolic contrast agents in the MCAO mouse model. Both substrates were tested at a similar dose (1 mmol/kg) for neuroprotection. We observed that the interconversion between lactate and pyruvate was lower (metabolite ratios) or slower (kinetic rates) in stroke animals, regardless of whether it was HP [1‐^13^C] pyruvate or HP [1‐^13^C] lactate given as a bolus. The lower conversion rates after stroke suggest that either the permeability across the BBB of both probes was reduced and/or the LDH activity was globally decreased. A point to consider is that due to cerebral ischemia a significant proportion of brain tissue loses temporarily or permanently its functions. Similarly, metabolic processes may also be affected, and the reduced rates observed may reflect, at least partly, a reduced volume of metabolically active neural tissue. Previous studies reported that the expression of monocarboxylate transporters (MCTs) was increased and their distribution modified after stroke [19, 62]. Consequently, one would anticipate that the HP monocarboxylates injected as a bolus would be more accessible to brain cells, which would be reflected in higher and faster conversion of lactate and pyruvate, respectively. Interestingly, here we observed the reverse behavior. Although there are more “entry points” for the substrate, the increased expression of transporters does not guarantee an increased efficacy (activity). Moreover, as the directionality of transport through MCTs is determined by the substrate and proton gradients, the rise in the endogenous lactate concentration [57] after stroke may interfere and reduce the amount of monocarboxylates that are able to cross the BBB, manifested here by a reduced and slower downstream conversion. This highlights the unique information provided by HP tracers that is tightly related to the actual efficacy of biochemical processes [63] and how MRS of HP probes is sensitive to fine changes in LDH activity [47].

When comparing the conversion of the HP substrates into alanine and bicarbonate after stroke, one should recall that according to our model, in HP [1‐^13^C] lactate experiments, similar to healthy animals, only the production of pyruvate involves lactate transport across the BBB [18] combined with enzymatic conversion, while the subsequent steps are considered intracellular. This leads to interesting observations: the production of bicarbonate comprises two distinct biochemical steps: first, the conversion of lactate to pyruvate, and second, the conversion of pyruvate to bicarbonate. In the kinetic analysis, the initial step is slower in stroke animals compared to sham, likely due to the reasons mentioned in the previous paragraph, while the second step appears faster. Note that the second step stems from a smaller ^13^C labeled pool of pyruvate in stroke mice compared to healthy mice (Figure 4D). The metabolite ratio analysis showed that bicarbonate production from HP lactate tended to be reduced (cBLR) after stroke. Hence, altogether, it suggests that the initial step (*k*_LP_) is rate‐limiting, and once pyruvate is produced, it can rapidly enter the TCA cycle (*k*_PB_). A limitation of this study is the small sample size, which may prevent finding significant changes in measurements that are close to the detection limit and have high variability. This is particularly noticeable in measurements like those of bicarbonate and, indeed, power analysis indicated that this study was underpowered to detect any difference, and a larger sample size is needed (*n* = 9 per group). Moreover, the observed trends might become significant if measured in larger animals, where not only bicarbonate, but other signals too, are inherently stronger.

Further metabolic differences were observed in the [1‐^13^C] alanine labeling from both substrates, with the alanine‐to‐pyruvate or alanine‐to‐lactate ratio being lower after stroke compared to sham. Although more investigations are needed to validate that the observed alanine was produced in the brain, as alanine aminotransferase levels are low in the healthy brain [64], the lower HP alanine label in stroke animals may be explained by the steadily elevated endogenous alanine pool size in acute ischemic stroke [19, 57]. Interestingly, the kinetic analysis after HP pyruvate injection did not indicate any change in the dynamics of alanine at any time point, while in HP lactate experiments, a trend towards faster *k*_PA_ was observed in MCAO 1 h animals compared to shams. The labeling of alanine is composed of two steps in the model: first, the production of pyruvate from lactate, and second, the transamination to alanine. Similarly, as above, the initial step (*k*_LP_) is rate‐limiting in the production of alanine in MCAO 1 h animals; therefore, even though the second step (*k*_PA_) appears faster in MCAO 1 h, this is coherent with the lower alanine‐to‐lactate ratio observed in these animals.

When evaluating the linear correlation between the model‐free analysis (metabolite ratios) and kinetic analysis (metabolic modeling), we found that in HP pyruvate experiments, only a weak correlation was seen in most comparison pairs, namely, metabolite ratios and the corresponding kinetic rate of the metabolic transformation between the metabolites. The only strong correlation was between the lactate‐to‐pyruvate ratio and the pyruvate‐to‐lactate rate constant in HP [1‐^13^C] pyruvate experiments, providing equivalent information as previously reported [55]. For the remaining HP pyruvate comparison pairs, only a weak correlation was found.

In HP lactate experiments, the correlation between the model‐free analysis and kinetic analysis revealed moderate dependence on all ratio and kinetic rates pairs. This indicates that for the same biochemical processes, the results generated by each method are similar but not equivalent. It is important to highlight that the kinetic analysis revealed more convincing *p*‐value differences between healthy and stroke animals than the ratio analysis.

To summarize, here we demonstrate the feasibility of quantifying the global cerebral metabolic kinetics of HP [1‐^13^C] pyruvate and HP [1‐^13^C] lactate used at reported protective doses in both healthy animals and in mice subjected to ischemia–reperfusion. Both substrates showed distinct global cerebral metabolism between healthy and ischemic brains. Major changes took place in the first 2 h after reperfusion, reflecting the damage as well as the metabolic reprogramming set in motion to meet the energetic demands after blood flow is restored in the acute phase of stroke. Although HP lactate metabolism is more difficult to measure due to the lower polarization and magnitude of the conversion into downstream metabolites compared to HP pyruvate, it achieved similar kinetic contrasts. Unlike HP pyruvate, measuring the metabolism of HP lactate offers the possibility of distinguishing between uptake and metabolism since subsequent steps can be measured. On the biological side, the concentration of metabolite administered as a potential therapy remains closer to the physiological concentration of lactate than that of pyruvate, and lactate is currently better characterized for neuroprotective effects, being already tested in phase 2 clinical trials for acute brain injuries [42, 65]. Overall, while pyruvate is better established as an imaging probe [12, 13, 66], lactate might be advantageous on the therapeutic side. However, further studies are needed, perhaps in more favorable animal models or in humans, to allow localized evaluation of HP lactate metabolism in challenging conditions, especially in terms of the signal‐to‐noise ratio and spatial resolution gained from mouse models. Despite the global measurements assessing the metabolism of the whole brain, which combines healthy tissue, potentially salvageable penumbra, and the core of the focal ischemic injury, this study prepares the ground for further investigation to fully exploit the potential of HP pyruvate and lactate as metabolic contrasts for stroke theranostics.

## 5 Conclusion

Metabolic MRI following the injection of HP tracers has been explored in many diseases, even at the clinical level, for more than a decade. To date, pyruvate is the most used tracer. Lactate has been studied as a neuroprotective treatment in the context of stroke and can be HP. In this study, we demonstrate the feasibility of hyperpolarizing and injecting lactate at reported protective doses and compare its performance with that of the gold standard HP pyruvate. While HP lactate still suffers from technical and physical limitations that require optimization of the hyperpolarization and detection schemes, our results suggest that HP lactate is worth exploring as a theranostic biosensor for stroke and may help to clarify the mechanism of lactate neuroprotection.

## Supporting information


**Figure S1:** Schematic of cerebral lactate and pyruvate metabolism.
**Figure S2:** Forward kinetic rates with models having reversible LDH conversion.
**Figure S3:** Kinetic rates as a function of T_1_.
**Figure S4:** Non‐normalized metabolite ratios.
**Figure S5:** Non‐normalized and elimination kinetic rate constants following HP pyruvate injection.
**Figure S6:** Non‐normalized and elimination kinetic rate constants following HP lactate injection.
**Figure S7:** Concentrations of selected metabolites in individual animals.
**Figure S8:** Typical proton spectra acquired in animals receiving HP tracer bolus after reperfusion.
**Figure S9:** Comparison of metabolite concentrations in MCAO mice with and without HP bolus.

## Data Availability

The data that support the findings of this study are available on request from the corresponding author. The data are not publicly available due to privacy or ethical restrictions.
