# Supplementary material for: Tracking Hyperpolarized [1‐13C] Pyruvate and [1‐13C] L‐Lactate Metabolism in the Healthy and Post‐Stroke Mouse Brain
Source: NMR Biomed. 2025 Jul 6;38(8):e70094. doi: 10.1002/nbm.70094 (PMC12230207; doi:10.1002/nbm.70094)
Supplement: Supplementary file 1 — Figure S1: Schematic of cerebral lactate and pyruvate metabolism. Figure S2: Forward kinetic rates with models having reversible LDH conversion. Figure S3: Kinetic rates as a function of T1. Figure S4: Non‐normalized metabolite ratios. Figure S5: Non‐normalized and elimination kinetic rate constants following HP pyruvate injection. Figure S6: Non‐normalized and elimination kinetic rate constants following HP lactate injection. Figure S7: Concentrations of selected metabolites in individual animals. Figure S8: Typical proton spectra acquired in animals receiving HP tracer bolus after reperfusion. Figure S9: Comparison of metabolite concentrations in MCAO mice with and without HP bolus. [file NBM-38-e70094-s001.docx]

**Tracking hyperpolarized [1-^13^C] pyruvate and [1-^13^C] L-lactate metabolism in the healthy and post-stroke mouse brain**

Lê Thanh Phong^1#^, Buscemi Lara^2,3 #^, Lepore Mario^4^, Vinckenbosch Elise^1^, Lanz Bernard^4,5^, Gruetter Rolf^1^, Hirt Lorenz^2,3^, Hyacinthe Jean-Noël^1,6 + *^, Mishkovsky Mor^1 +*^

^1^ Laboratory of Functional and Metabolic Imaging, École Polytechnique Fédérale de Lausanne (EPFL), Lausanne, Switzerland

^2^ Department of Clinical Neurosciences, Lausanne University Hospital (CHUV), Lausanne, Switzerland

^3^ Department of Fundamental Neurosciences, University of Lausanne (UNIL), Lausanne, Switzerland

^4^ CIBM Center for Biomedical Imaging, Lausanne, Switzerland.

^5^ Animal Imaging and Technology, École Polytechnique Fédérale de Lausanne (EPFL), Lausanne, Switzerland.

^6^ Image Guided Intervention Laboratory, Faculty of Medicine, University of Geneva, Geneva, Switzerland.

^#^ Authors contributed equally to this work.

^+^ Authors contributed equally to this work.

*** Correspondence:**

Mor Mishkovsky

# Supplementary information

## Figure S1: Schematic of cerebral lactate and pyruvate metabolism


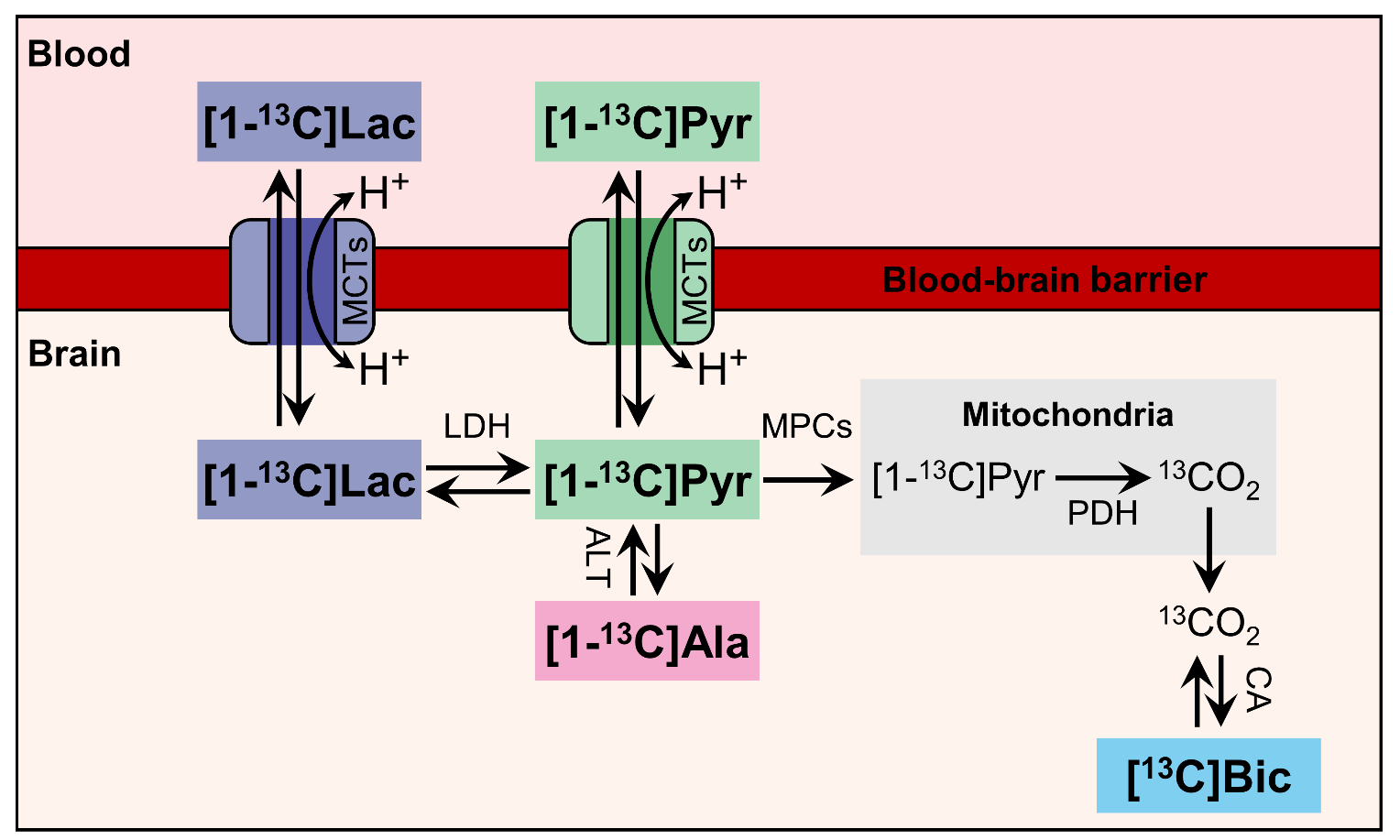


Simplified schematic of cerebral transport and metabolism of HP [1-^13^C] pyruvate and HP [1-^13^C] lactate. Both substrates can cross the blood-brain barrier (BBB) via monocarboxylate transporters (MCTs). Intracellular [1-^13^C] lactate and [1-^13^C] pyruvate pool inter-conversion step via lactate dehydrogenase (LDH). [1-^13^C] pyruvate is either converted into [1-^13^C] alanine by alanine aminotransferase (ALT) or transported into the mitochondria via mitochondrial pyruvate carriers (MPCs), then oxidized by pyruvate dehydrogenase (PDH), producing ^13^CO_2_ remaining in equilibrium with [^13^C] bicarbonate via carbonic anhydrase (CA).

## Figure S2: Forward kinetic rates with models having reversible LDH conversion.


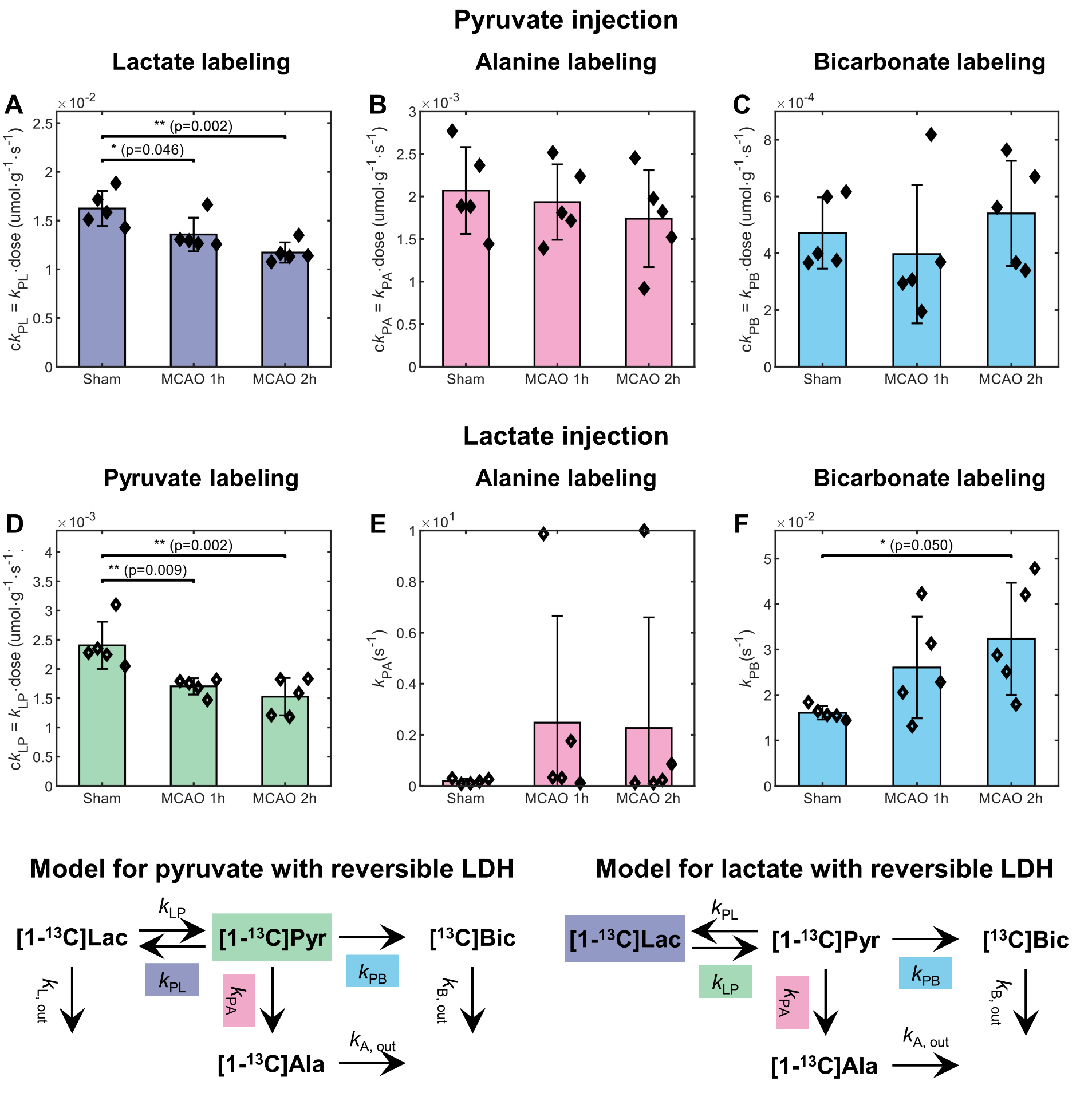


Kinetic rates following injection of HP [1-^13^C] pyruvate (**A-C**) or HP [1-^13^C] lactate (**D-F**) using metabolic models taking the reversible LDH conversion into account (lower panel). Data are displayed as the mean ± standard deviation and overlaid with individual data points (circles: sham, squares: MCAO 1h, diamonds: MCAO 2h, filled shapes: pyruvate injection, hollow shapes: lactate injection). Normalized rate constants of pyruvate-to-lactate (c*k*_PL_, **A**), pyruvate-to-alanine (c*k*_PA_, **B**), pyruvate-to-bicarbonate (c*k*_PB_, **C**), lactate-to-pyruvate (c*k*_LP_, **D**) conversion. Rate constants of pyruvate-to-alanine (*k*_PA_, **E**) and pyruvate-to-bicarbonate (*k*_PB_, **F**) turnover. The forward kinetic rates calculated are similar to those calculated with the forward-only models. Therefore, for simplicity, we considered the forward-only models in the main paper.

## Figure S3: Kinetic rates as a function of T_1_


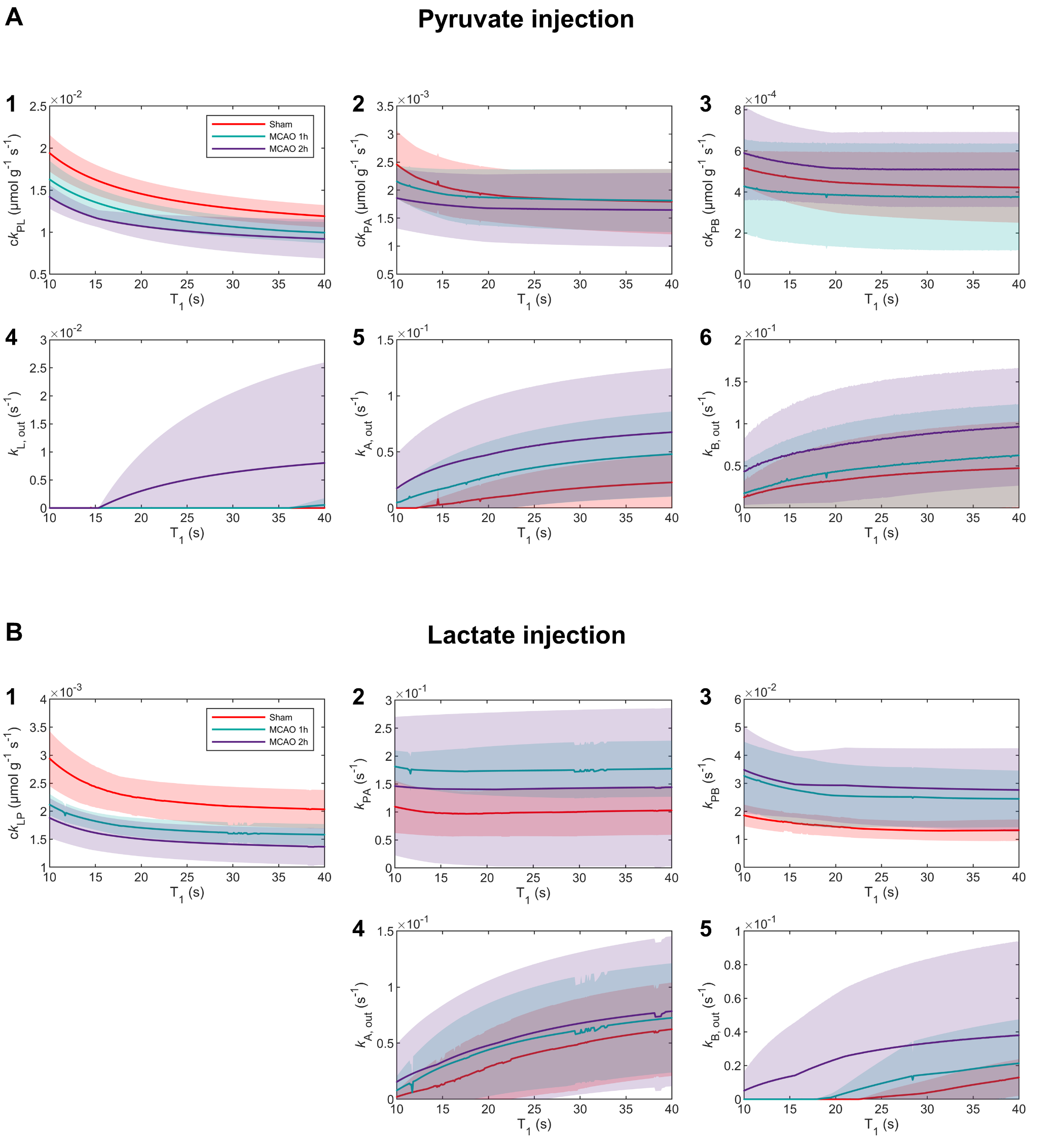


Kinetic rate constants computed from the pyruvate model (**A**) and the lactate model (**B**) for a T_1_ parameter ranging from 10 to 40 s by 0.1 s increments. For each group, the mean value is represented by the solid line, and the standard deviation of the mean across the animals is represented by the shaded areas. Normalized kinetic rates of pyruvate-to-lactate (c*k*_PL_, **A.1**), pyruvate-to-alanine (c*k*_PA_, **A.2**), pyruvate-to-bicarbonate (c*k*_PB_, **A.3**) as well as kinetic rates of lactate elimination (*k*_L,out_, **A.4**), alanine elimination (*k*_A,out_, **A.5**) and bicarbonate elimination (*k*_B,out_, **A.6**) following an HP [1-^13^C] pyruvate injection. Normalized kinetic rate of lactate-to-pyruvate (c*k*_LP_, **B.1**), and kinetic rates of pyruvate-to-alanine (*k*_PA_, **B.2**), pyruvate-to-bicarbonate (*k*_PB_, **B.3**), alanine elimination (*k*_A,out_, **B.4**) and bicarbonate elimination (*k*_B,out_, **B.5**) following an HP [1-^13^C] lactate injection. Overall, the computed kinetic rates vary smoothly as a function of T_1_, therefore the kinetic models are stable regarding this parameter. The differences between groups are globally kept as T_1_ is changed, except for c*k*_PA_ following a pyruvate injection, with similar values for sham and MCAO 1h for T_1_ above 20 s (**A.2**), and for *k*_PB_ after a lactate injection where we observe a greater difference between both MCAO groups with T_1_ values above 15 s (**B.3**). As the T_1_ parameter increases, the reduced longitudinal magnetization losses are compensated by an increase in all elimination rate constants (**A.4-6** and **B.4-5**).

## Figure S4: Non-normalized metabolite ratios


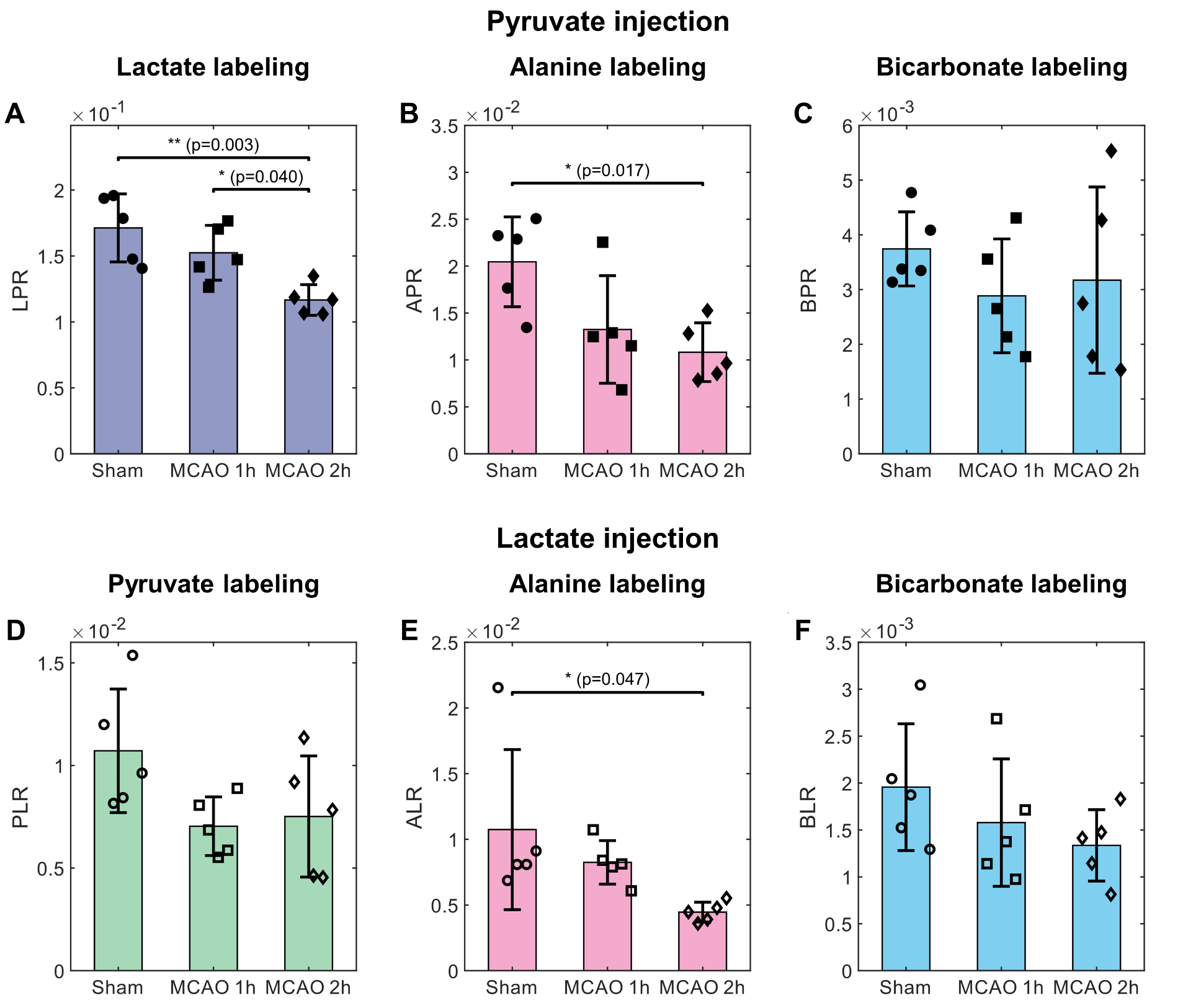


Non-normalized metabolite ratios for the experiments with an HP [1-^13^C] pyruvate injection **(A-C)** and experiments with an HP [1-^13^C] lactate injection **(D-F)**. Lactate-to-pyruvate ratio (LPR, **A**), alanine-to-pyruvate ratio (APR, **B**), bicarbonate-to-pyruvate ratio (BPR, **C**), pyruvate-to-lactate ratio (PLR, **D**), alanine-to-lactate ratio (ALR, **E**), bicarbonate-to-lactate ratio (BLR, **F**).

## Figure S5: Non-normalized and elimination kinetic rate constants following HP pyruvate injection


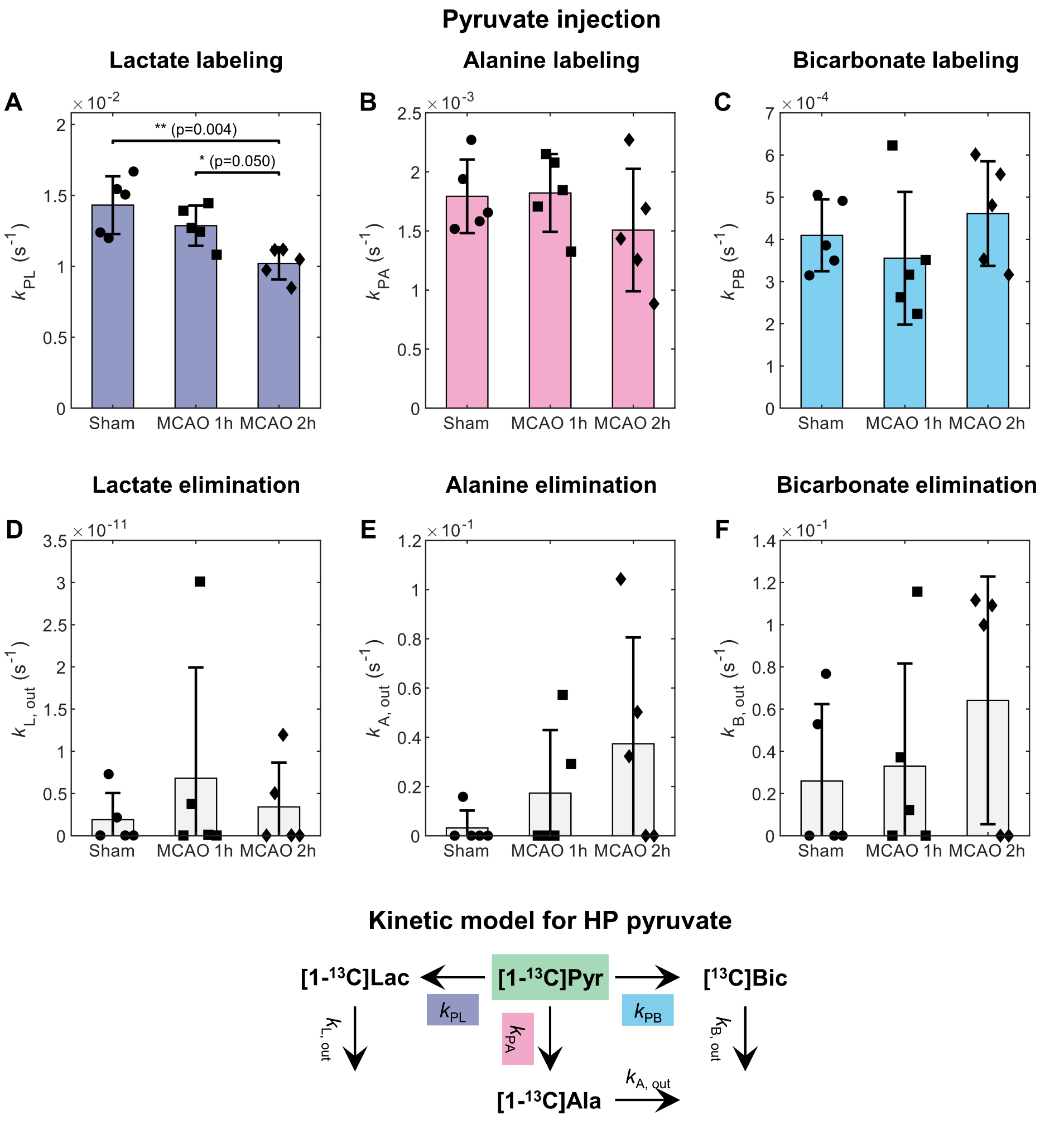


Kinetic rate constants of pyruvate-to-lactate (*k*_PL_, **A**), pyruvate-to-alanine (*k*_PA_, **B**), pyruvate-to-bicarbonate (*k*_PB_, **C**), lactate elimination (*k*_L,out_, **D**), alanine elimination (*k*_A,out_, **E**), bicarbonate elimination (*k*_B,out_, **F**) following an HP [1-^13^C] pyruvate injection.

## Figure S6: Non-normalized and elimination kinetic rate constants following HP lactate injection


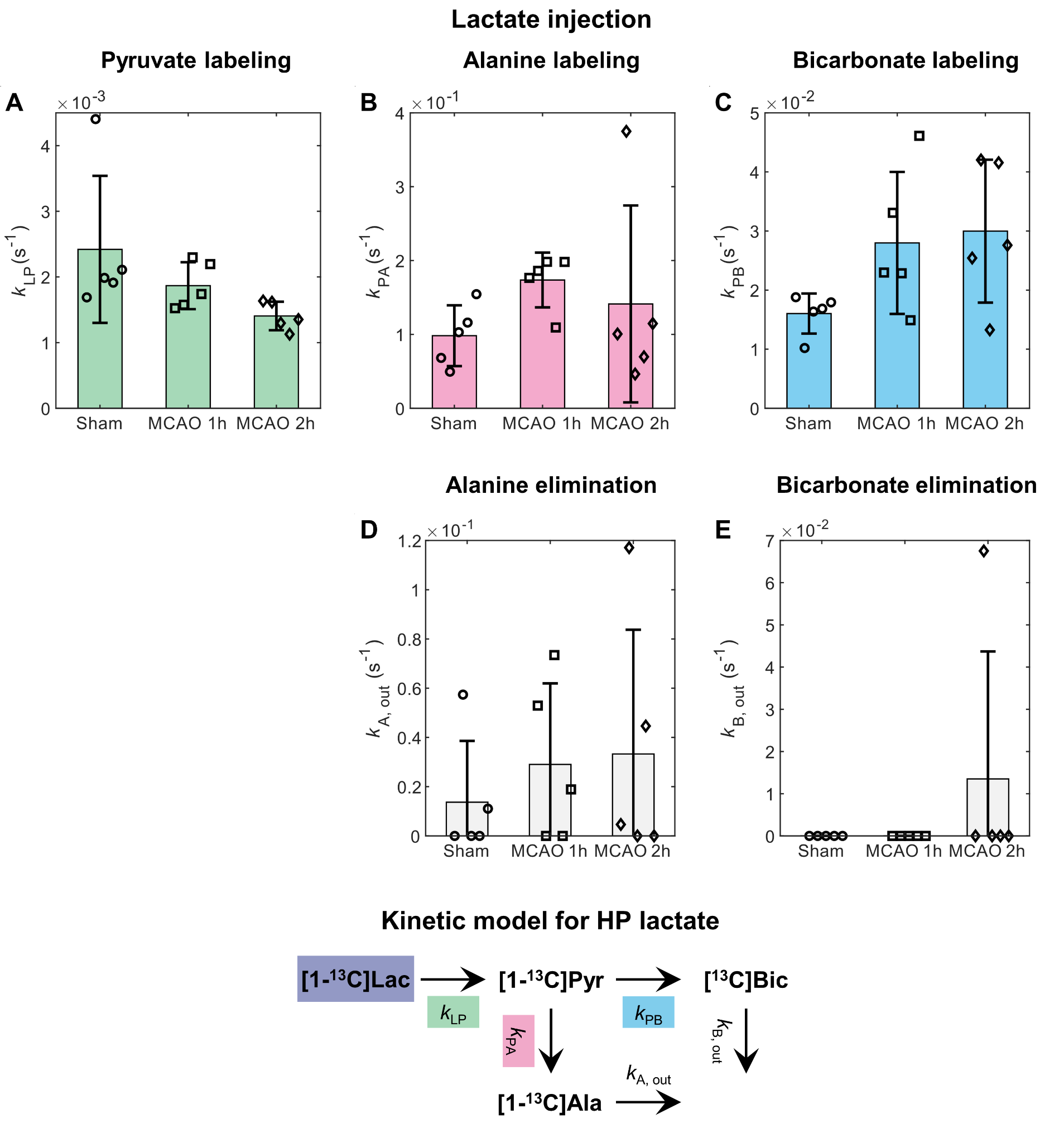


Kinetic rate constants of lactate-to-pyruvate (*k*_LP_, **A**), pyruvate-to-alanine (*k*_PA_, **B**), pyruvate-to-bicarbonate (*k*_PB_, **C**), alanine elimination (*k*_A,out_, **D**), bicarbonate elimination (*k*_B,out_, **E**) following an HP [1-^13^C] lactate injection.

## Figure S7: Concentrations of selected metabolites in individual animals


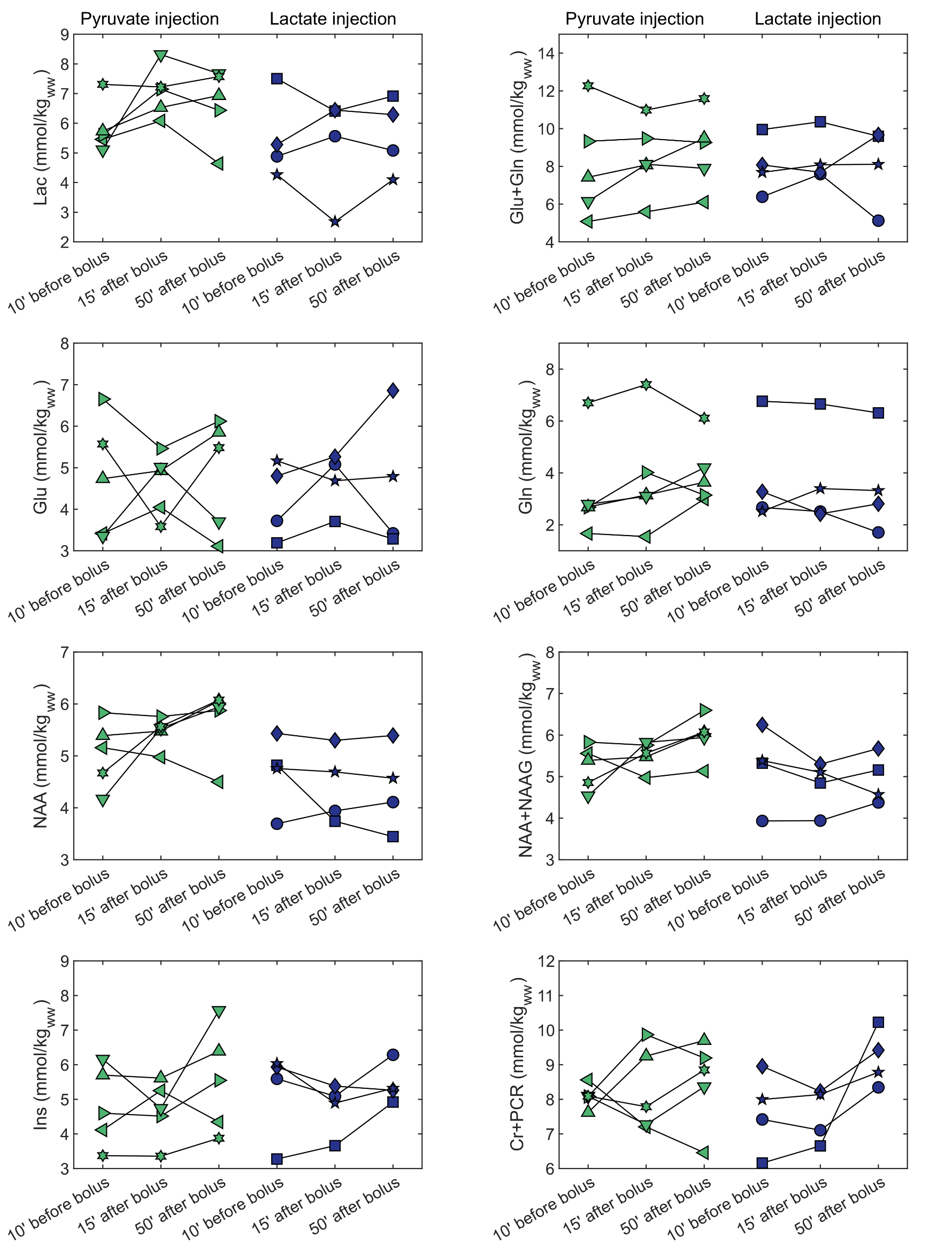


Concentrations of selected metabolites detected in sham animals at 10 min before injection, 15 min after injection and 50 min after injection in experiments with HP [1-^13^C] pyruvate (green datapoints) and [1-^13^C] lactate (blue datapoints). The lines relate datapoints from the same animal. No significant difference was observed between the compared timepoints. Abbreviations: creatine (Cr), myo-inositol (Ins), glutamine (Gln), glutamate (Glu), lactate (Lac), N-acetyl-aspartate (NAA), N-acetyl-aspartyl-glutamate (NAAG), phosphocreatine (PCr).

## Figure S8: Typical proton spectra acquired in animals receiving HP tracer bolus after reperfusion.


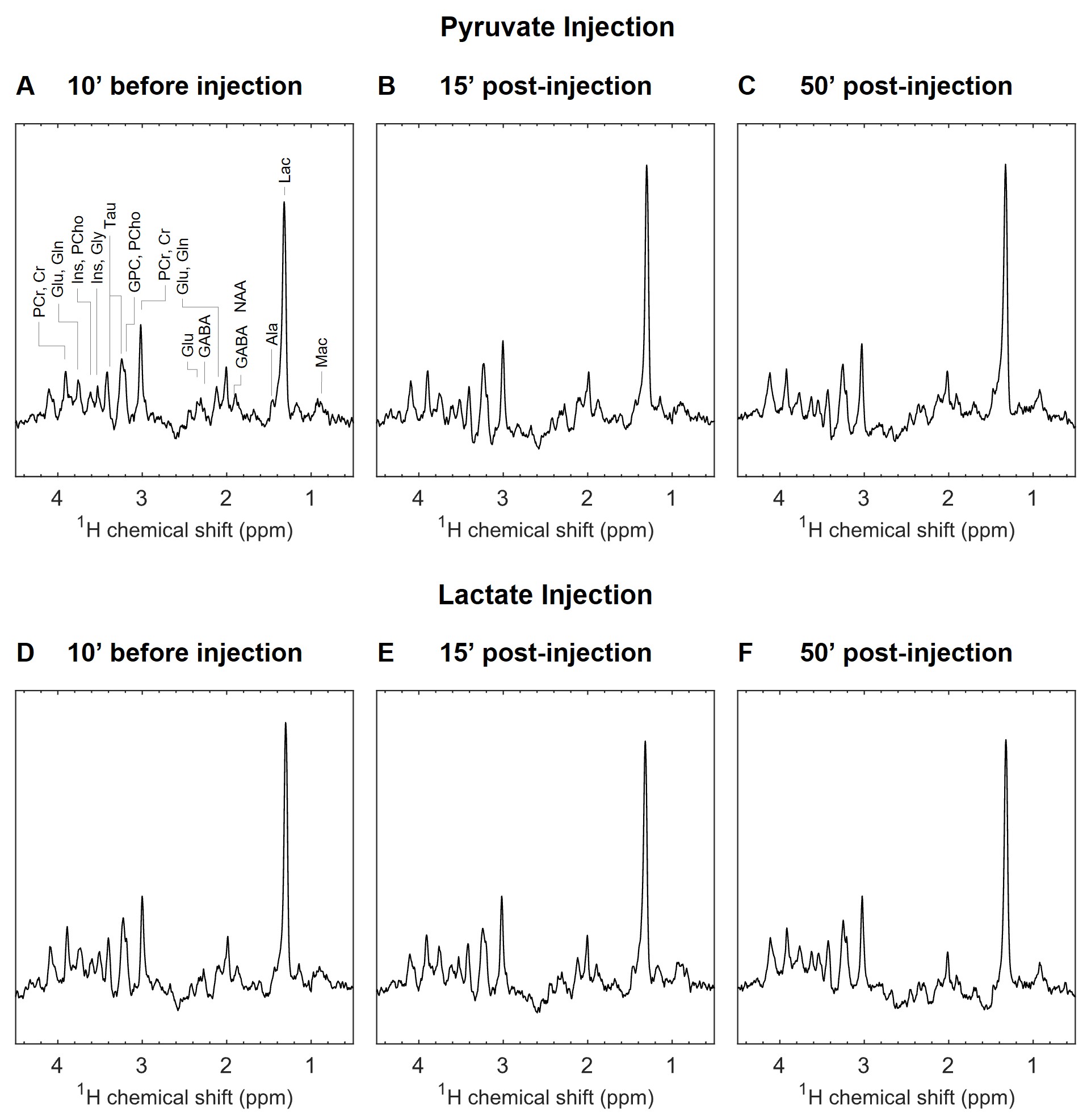


Representative ^1^H MRS spectra (160 averages, 640 s of acquisition time for each point) of MCAO mice that received HP pyruvate bolus (top panel) or HP lactate bolus (bottom panel) after reperfusion. A and D show spectra acquired before bolus administration. B and C were acquired approximately 10 min after HP bolus injection, and C and F at 50 min after HP bolus injection.

## 9.9 Figure S9: Comparison of metabolite concentrations in MCAO mice with and without HP bolus


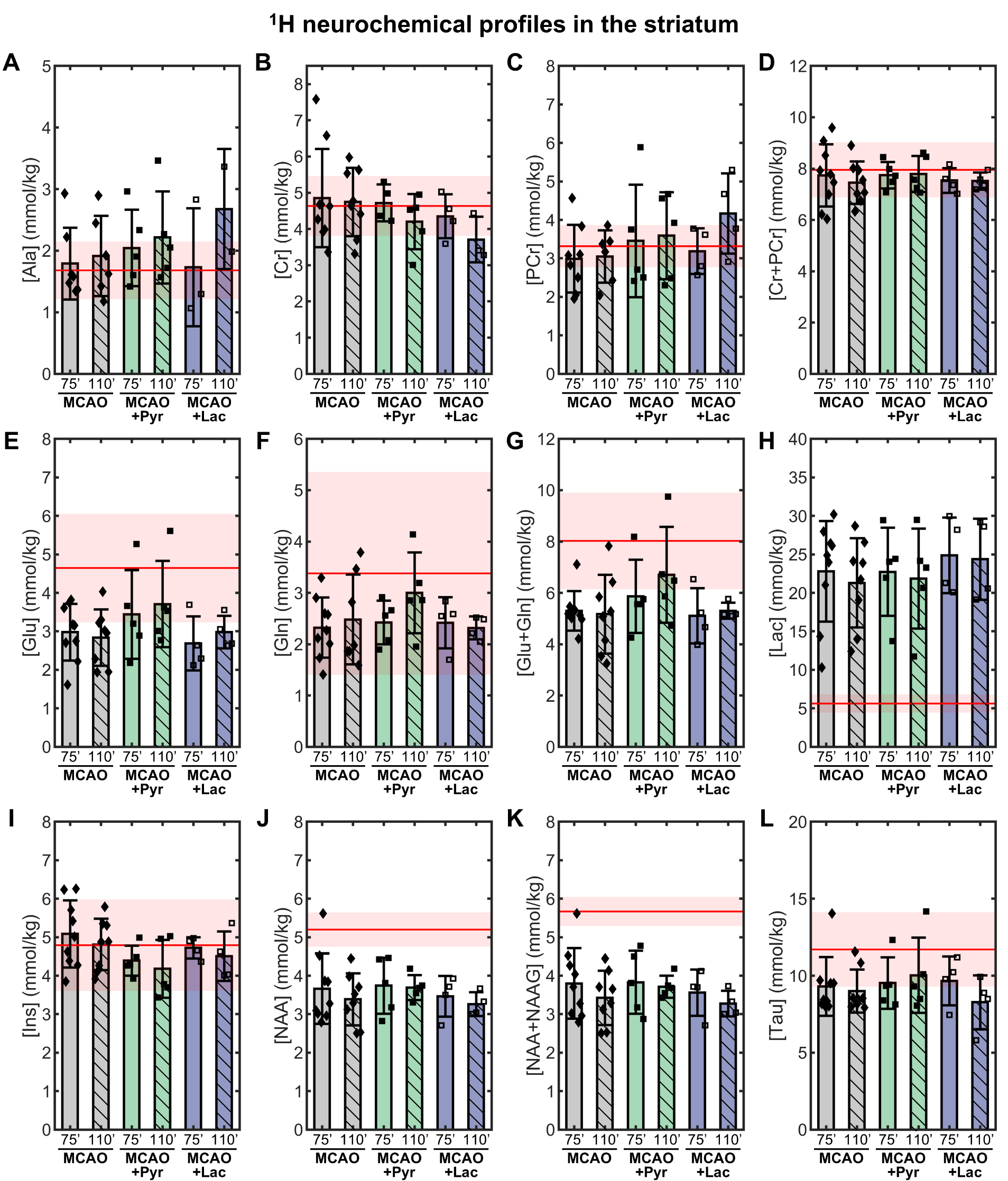


Concentrations of selected metabolites measured at 75 min (full bars) and 110 min (full and dashed bars) after reperfusion in MCAO mice. The 75 min and 110 min correspond to about 15 min and 50 min, respectively, after the injection of either HP pyruvate (green) or lactate (purple). MCAO mice that did not receive any injection appear in gray. Data are displayed as the mean ± standard deviation and overlaid with individual data points. The red line represents the average metabolite concentration value quantified in the sham operated animals with the SD designated by the red shading. No significant differences were observed between all six MCAO groups.
